# *In vitro* fermentation characteristics of dietary fibers using fecal inocula from dogs fed a canned diet and treated with metronidazole

**DOI:** 10.3389/fvets.2025.1670624

**Published:** 2025-10-29

**Authors:** Sara E. Martini, Patrícia M. Oba, Elizabeth L. Geary, Laura L. Bauer, Ryan N. Dilger, Kelly S. Swanson

**Affiliations:** ^1^Department of Animal Sciences, University of Illinois Urbana-Champaign, Urbana, IL, United States; ^2^Division of Nutritional Sciences, University of Illinois Urbana-Champaign, Urbana, IL, United States; ^3^Department of Veterinary Clinical Medicine, University of Illinois Urbana-Champaign, Urbana, IL, United States

**Keywords:** antibiotic, canine microbiome, fiber fermentation, gastrointestinal health, pet health

## Abstract

**Introduction:**

Metronidazole is a potent antibiotic often prescribed to treat gastrointestinal enteropathies; however, it is known to induce loose stools, negatively alter the fecal microbiome, and affect fecal metabolite production. Dietary intervention may aid in post-antibiotic recovery; however, little research has been conducted on the potential of fiber utilization for microbial recovery in canines.

**Methods:**

Using an *in vitro* fermentation assay, the objective of this study is to investigate the fermentation characteristics of dietary fibers using fecal inocula from dogs treated with metronidazole. Four healthy male beagles (age = 1.62 ± 0.02 year) were fed a commercial canned diet for 2 weeks, then administered metronidazole (20 mg/kg BW BID) for 2 weeks. Fresh fecal samples were collected at weeks 2 (before antibiotic treatment; ABX−) and 4 (after antibiotic treatment; ABX+), stabilized in a 20% glycerol solution, and then frozen. On the day of *in vitro* fermentation, feces from each time point were thawed and used to inoculate the tubes. At baseline and after 6, 12, and 18 h of fermentation, pH, short-chain fatty acids (SCFA), and microbiota were measured. Blank-corrected changes from the baseline data were analyzed using repeated measures and the MIXED procedure in SAS 9.4, with significance set at a *p* value <0.05.

**Results:**

Pectin fermentation reduced (*p* < 0.001) pH and increased (*p* < 0.001) SCFA over time, but the responses were lower (*p* < 0.001) in ABX+ than in ABX−. Beet pulp fermentation also reduced (*p* < 0.001) pH and increased (*p* < 0.001) SCFA over time. The pH change was small between inoculum sources, but SCFA were different (*p* < 0.001) between ABX+ and ABX−. Chicory pulp fermentation reduced (*p* < 0.001) pH over time, with greater (*p* < 0.01) reductions in ABX+ than in ABX−. Chicory pulp fermentation increased SCFA but had different patterns depending on the inoculum source. Metronidazole altered microbiota populations by reducing bacterial alpha diversity (*p* < 0.001). Analysis of bacterial beta diversity revealed separate clusters in dogs based on metronidazole administration. Beta diversity analysis also showed that tubes containing chicory pulp clustered separately from those containing other fibers. The relative abundance of over 50 bacterial genera differed (*p* < 0.05) among the inoculum sources.

**Discussion:**

In summary, interesting fermentation patterns were observed in response to varying fiber sources, allowing for improved insights into their potential abilities in antibiotic-treated dogs.

## Introduction

Canine gastrointestinal (GI) ailments are among the most common reasons for visiting veterinary clinics, presenting as diarrhea, vomiting, anorexia, and/or lethargy. While the root causes of these issues might be various factors (e.g., age, health status, drug usage, sudden dietary change, and environmental stressors), antibiotics are among the first-line regimens for treatment. In a recent study performed by Weese et al. (1), antimicrobials were prescribed in 85% (702,576 patient visits) of canine visits and 15% (128,441 patient visits) of feline visits using data collected across 1,084 clinics in the United States. The selection of antimicrobials in treatment plans may be influenced by many factors (e.g., host digestive and metabolic issues and pathogen infection); however, this analysis demonstrated that bactericidal agents were the top choice for dogs [cefpodoxime (29%), amoxicillin-clavulanate (22%), and metronidazole (21%)] and cats [cefovecin (43%) and amoxicillin-clavulanate (35%)] (1). Similar results were reported in a previous analysis of veterinary clinics, where 72% of the antibiotics prescribed were bactericidal agents [penicillins and fluoroquinolones; (2)]. In both studies, antimicrobial prescriptions targeted the GI tract, respiratory tract, urinary tract, and skin (1, 2). Although these studies highlight the prolific use of bactericidal agents, they also emphasize the need for more refined guidelines for administration to combat the evolving epidemic of antimicrobial resistance in veterinary and human medicine.

Metronidazole is commonly used for Giardia or *Clostridium perfringens* infections in small animal veterinary medicine (3, 4), but research has demonstrated significant negative alterations in fecal scores (stool firmness), reductions in microbial populations, bacterial diversity, and microbial-derived metabolites [e.g., short-chain fatty acids (SCFAs), bile acids, fatty acids, and sterols], and increases in fecal bacterial dysbiosis measures in dogs and cats (3, 5–10). As summarized in a recent review by Shah et al. (11), disruption of the GI microbiome induced by antibiotics can negatively affect host and microbial functions, as many of these commensal bacterial taxa are involved in SCFA production, including *Bacteroides, Blautia, Faecalibacterium, Megamonas, Prevotella, Ruminococcus, Turicibacter,* and *Eubacterium,* and are depleted with antibiotic usage, as previously demonstrated (5–7, 12–14). Therefore, the restoration of these bacteria is crucial to ensure recovery, a process that may be aided by dietary treatments.

Antibiotic usage can be highly effective, but overuse or repeated administration can lead to negative and potentially long-term consequences. To combat some of these effects, the application of functional ingredients (e.g., dietary fiber) may be of interest in veterinary medicine. Dietary fibers are commonly used to support GI health in companion animals; however, fiber sources can vary based on their physicochemical properties (e.g., solubility, fermentability, viscosity, and water-holding capacity) and physiological effects (e.g., metabolic regulation, fecal bulking, fecal frequency, immune function, and microbiome composition/function) within the host (11, 15, 16). The physicochemical properties of fiber can also play a prominent role in processing, as more soluble and viscous fibers may be utilized in commercial canned or high-moisture diets as binding agents (e.g., gums), whereas insoluble fibers may be used in weight loss diets to contribute to satiety and diet bulking.

Non-fermentable, insoluble, and non-viscous fibers (e.g., cellulose) are not well utilized by microbes, whereas highly fermentable and soluble fibers (e.g., pectin, gums) are rapidly fermented by microbes, leading to the production of SCFA that may be used by host colonocytes or other microbes. Commercial diets often have variable inclusions of both insoluble and soluble fiber fractions, but the ratio can be adjusted based on animal needs. For example, diets with greater inclusion of insoluble fibers may be advantageous for animals with constipation, as these fibers can increase fecal bulk and reduce transit time. However, moderately fermentable fibers (e.g., beet pulp and chicory pulp) may have other beneficial effects in companion animals. Pulps are common byproducts of the human food industry and can be beneficially used in pet food because they consist of several insoluble and soluble fibers (e.g., hemicelluloses, cellulose, and pectin) (17, 18). Beet pulp (63.0% total dietary fiber; 54.2% soluble fiber) and chicory pulp (70.4% total dietary fiber; 46.1% soluble) have previously been demonstrated to have great fermentation potential and lead to increased SCFA production when fermented *in vitro* (17). In pet food formulations, a balance between insoluble and soluble fibers has been suggested. Fiber fraction ratios can be modified to favor certain microbial (e.g., shifts to favor SCFA-producing bacteria) or metabolic (e.g., bowel movement frequency) outcomes and may prove beneficial in cases of diet-related GI issues.

Considering the many negative side effects of metronidazole administration and the previously reported benefits associated with dietary fiber consumption, the objective of this study is to investigate the fermentation characteristics of dietary fibers using fecal inocula from dogs fed a canned diet and treated with metronidazole. Based on previous research and the variety of physicochemical properties and fermentability, cellulose, pectin, beet pulp, and chicory pulp were selected as fermentation substrates. Cellulose served as a negative control (low fermentation), pectin was used as a positive control (high fermentation), and beet pulp and chicory pulp were used as moderately fermentable test fibers. We hypothesized that fecal inoculum from dogs treated with antibiotics (ABX+) would have lower microbial diversity and would negatively influence the fermentation rate and metabolite production compared to fecal inoculum collected prior to metronidazole administration. Of the substrates utilized, we hypothesized that pectin would have the highest fermentability, beet pulp would be more fermentable than chicory pulp, and cellulose would be the least fermentable. Lastly, we hypothesized that SCFA concentrations would increase as fermentability increased, ultimately favoring the abundance of SCFA producers (e.g., *Blautia, Bacteroides, Turicibacter, Lactobacillus*, and *Bifidobacterium*).

## Materials and methods

All animal procedures were approved by the University of Illinois Institutional Animal Care and Use Committee prior to experimentation (IACUC #23070).

### Animals, diets, and experimental design

Four healthy adult male beagles (mean age = 1.62 ± 0.02 year; mean body weight = 8.63 ± 0.59 kg) were used to collect fresh fecal samples to be used as a source of inoculum. All dogs were housed individually in an environmentally controlled facility at the University of Illinois Urbana-Champaign. Despite individual housing, dogs had constant access to toys and were socialized at least twice per week, which provided them with the ability to socialize with humans and other dogs. Dogs had free access to fresh water at all times and were fed twice daily (8 a.m. and 3 p.m.). All dogs were fed a commercial canned diet (Pedigree Chopped Ground Dinner with Chicken; Pedigree Petfoods, McLean, VA, USA) formulated to meet all nutrient recommendations for adult dogs at the maintenance provided by the Association of American Feed Control Officials (19) to maintain body weight. Food offered and refused was measured daily to calculate intake, and any observations of vomiting or negative reactions were recorded. Dogs were weighed, and body condition scores were assessed using a 9-point scale (20) once a week prior to morning feeding throughout the study.

The study was 4 weeks long. The study started with a 2-week baseline in which all dogs consumed the diet only. After baseline, dogs received metronidazole (Metronidazole Compounded Oil Liquid Chicken Flavored; Chewy, Inc.; Boston, MA, USA) at a dosage of 20 mg/kg orally twice daily (mealtimes) for 2 weeks. Fecal samples were collected at the end of baseline (week 2; ABX−) and antibiotic administration (week 4; ABX+) and stabilized in a 20% glycerol solution in duplicate. Briefly, a 10 g fecal aliquot was collected in a 50 mL conical tube with 10 mL of a 20% glycerol solution (21). Samples were then frozen at −80 °C until the *in vitro* fermentation study was conducted.

### *In vitro* fermentation assay

On the day of the *in vitro* experiment, fecal samples were carefully thawed and heated to 39 °C using a water bath, pooled by treatment (ABX− = pre-metronidazole inoculum collection; ABX+ = post-metronidazole inoculum collection), diluted 1:4 (wt/vol) in an anaerobic diluting solution, and blended for 15 s in a Waring blender (Waring Products, Stamford, CT, USA). Blended, diluted feces were filtered through 4 layers of cheesecloth and sealed in 125 mL serum bottles under a stream of CO_2_ to minimize exposure to oxygen. Sample and blank tubes were then aseptically inoculated with diluted feces and added to the medium (Table 1), as described by Bourquin et al. (22). A total of 4 mL of diluted feces was used to inoculate tubes containing 26 mL of semi-defined medium and one of the following fiber sources (300 mg/tube): cellulose (negative control), pectin (positive control), beet pulp, or chicory pulp.

**Table 1:** Composition of microbiological medium used in the *in vitro* experiment.

Component	Amount
Liquid solutions (mL/L)	
Solution A^a^	330.0
Solution B^b^	330.0
Distilled water	296.0
Water-soluble vitamin mix^c^	20.0
Trace mineral solution^d^	10.0
Folate/biotin solution^e^	5.0
Riboflavin solution^f^	5.0
Hemin solution^g^	5.0
Resazurin^h^	1.0
Short-chain fatty acid mix^i^	0.4
Solid chemicals, g in medium	
Yeast	0.5
Trypticase	0.5
Na_2_CO_3_	4
Cysteine hydrochloride	0.5

a Composition (g/L): NaCl, 5.4; KH_2_PO_4_, 2.7; CaCl_2_·H_2_O, 0.18; MgCl_2_·6H_2_O, 0.12; MnCl_2_·4H_2_O, 0.06; CoCl_2_·6H_2_O, 0.06; (NH_4_)_2_SO_4_, 5.4.

b Composition: K_2_HPO_4_, 2.7 g/L.

c Composition (mg/L): thiamin hydrochloride,100; D-pantothenic acid, 100; niacin, 100; pyridoxine, 100; p-aminobenzoic acid, 5; vitamin B_12_, 0.25.

d Composition (mg/L): EDTA (EDTA, disodium salt), 500; FeSO_4_·7H_2_O, 200; ZnSO_4_·7H_2_O, 10; MnCl_2_·4H_2_O, 3; H_3_PO_4_, 30; CoCl_2_·6H_2_O, 20; CuCl_2_·2H_2_O, 1; NiCl_2_·6H_2_O, 2; and Na_2_MoO_4_·2H_2_O, 3.

e Composition (mg/L): folic acid, 10; D-biotin, 2; NH_4_HCO_3_, 100.

f Hemin, 500 mg/L, in 10 mmol/L NaOH.

g Composition: riboflavin, 10 mg/L, in 5 mmol/L of 4-(2-hydroxyethyl)piperazine-1-ethanesulfonic acid, *N*-(2-hydroxyethyl)piperazine-*N′*-(2-ethanesulfonic acid) (HEPES).

h Resazurin, 1 g/L, in distilled H_2_O.

i Contained 250 μL/L each of *n*-valerate, isovalerate, isobutyrate, and *DL-α*-methylbutyrate.

In this experiment, highly fermentable pectin was used as a positive control, whereas cellulose served as a negative control because it has minimal fermentation potential. For the test substrates, moderately fermentable fibers consisting of beet pulp and chicory pulp, which are commonly used in the pet food industry, were used. Triplicate tubes of each fibrous substrate (~0.300 g/ tube) were incubated at 39 °C for 0, 6, 12, or 18 h with periodic mixing. At each time point, incubation was stopped, and samples were processed immediately. At each time point, the pH of the tube contents was measured using a pH meter. Samples to be analyzed (2 mL) for SCFA were mixed with 0.5 mL of 25% metaphosphoric acid and processed according to Erwin et al. (23) using a Hewlett-Packard (Avondale, PA, USA) Model 5890A gas chromatograph equipped with a flame ionization detector on a column (1.8 m × 4 mm i.d.) packed with GP 10% SP-1200/1% H_3_P0_4_ on 80/100 chromosorb WAW (Supelco, Bellefonte, PA, USA). The carrier gas was nitrogen, with a flow rate of 75 mL/min. The oven, injection port, and detector port temperatures were 125, 175, and 180 °C, respectively. Aliquots for microbial analyses were collected into sterile cryogenic vials and placed on dry ice until they were transferred to a − 80 °C freezer, where they were stored until analysis. Data were corrected using a blank tube (inocula and media, but no fiber source) and baseline (0 h) sample production.

### DNA extraction and MiSeq Illumina sequencing

Media collected after fermentation were centrifuged prior to extraction to improve the DNA extraction techniques. Approximately 1–1.5 mL of fermentation media was transferred into a microtube and centrifuged at 10,000 × *g* at 4 °C for 10 min (Eppendorf Centrifuge 5,424 R; Eppendorf Group, Hamburg, Germany). Supernatant was removed, and the pellet was transferred to PowerBead tubes provided in the DNeasy PowerLyzer PowerSoil Kit (MoBio Laboratories, Carlsbad, CA, USA) and further extracted according to the manufacturer’s protocol. Samples underwent bead beating using a vortex, followed by further centrifugation to purify the DNA, which was then quantified using a Qubit® 3.0 Fluorometer (Life Technologies, Grand Island, NY, USA). DNA quality was determined using an E-Gel Power Snap Electrophoresis Device (Invitrogen, Waltham, MA, USA) on E-Gel EX 1% Agarose Gels. The concentration of extracted DNA was quantified using a Qubit 3.0 Fluorometer (Life Technologies, Grand Island, NY, USA) and then submitted to the Roy J. Carver Biotechnology Center at the University of Illinois for Illumina sequencing with 16S rRNA gene amplicons that were generated using a Fluidigm Access Array (Fluidigm Corporation, South San Francisco, CA, USA) in combination with the Roche High Fidelity Fast Start Kit (Roche, Indianapolis, IN, USA). The primers 515F (5′-GTGCCAGCMGCCGCGGTAA-3′) and 806R (5′-GGACTACHVGGGTWTCTAAT-3′) that target a 252 bp fragment of the V4 region of the 16S rRNA gene were used for amplification (primers synthesized by IDT Corp., Coralville, IA, USA) (24). CS1 forward and CS2 reverse tags were added according to the Fluidigm protocol. Quality of the amplicons was assessed using a Fragment Analyzer (Advanced Analytics, Ames, IA, USA) to confirm amplicon regions and sizes. A DNA pool was generated by combining equimolar amounts of amplicons from each sample. The pooled samples were then size-selected on 1% agarose E-gel (Life Technologies, Grand Island, NY, USA) and extracted using a Qiagen gel purification kit (Qiagen, Valencia, CA, USA). Cleaned size-selected pooled products were run on an Agilent Bioanalyzer to confirm the appropriate profile and average size. Illumina sequencing was then performed on a MiSeq using v3 reagents (Illumina Inc., San Diego, CA, USA) at the Roy J. Carver Biotechnology Center at the University of Illinois.

### QIIME2 bioinformatics analysis

Forward reads were trimmed using the FASTX-Toolkit (version 0.0.14), and sequences were analyzed using QIIME 2.0 version 2023.7 (24). Raw sequence amplicons were imported into the QIIME2 package and analyzed using the DADA2 pipeline for quality control (QC value ≥ 20) (25). Samples were rarefied to 5,629 reads for cellulose-containing tubes, 11,533 reads for pectin-containing tubes, 11,860 reads for beet pulp-containing tubes, and 12,169 reads for chicory pulp-containing tubes. Subsequent samples were assigned to taxonomic groups using the SILVA database (SILVA 138; 99% OTU from the 515F/806R region of sequences, with the QIIME2 classifier trained on the 515F/806R V4 region of 16S) (26–28). Rarefied samples were used for alpha diversity and beta diversity. Principal coordinate analysis was performed using weighted and unweighted unique fraction metric (UniFrac) distances (29).

### Statistical analysis

Data were blank-corrected and analyzed using the Mixed Models procedure of SAS version 9.4 (SAS Institute Inc., Cary, NC, USA), with antibiotic treatment and time as fixed effects and the random effect of each replicate within each fiber. Normality was tested using the UNIVARIATE procedure in SAS. If data did not meet normality, they were analyzed using NPA1RWAY procedures, and Wilcoxon statistics were used to determine significance. The mean change from 0 h differences due to antibiotics, time, and antibiotic*time was determined using a Fisher-protected least significant difference with a Tukey adjustment to control for experiment-wise error. Statistical significance was set at a *p* value < 0.05, with tendencies set at a *p* value < 0.10.

## Results

### Baseline measures

All baseline (0 h) pH values and SCFA concentrations are presented in Supplementary Table 1 for tubes containing pectin, beet pulp, chicory pulp, or cellulose. The pH values were not affected by the inoculum source, but the SCFA concentrations were. In tubes containing pectin or beet pulp, butyrate concentrations were lower (*p* < 0.05) with the ABX+ inoculum. Acetate concentrations were higher (*p* < 0.05) with ABX− inoculum in tubes containing beet or chicory pulp. Tubes containing cellulose were not different at baseline, and propionate concentrations were not affected by the inocula.

All baseline blank-corrected bacterial phyla and genera relative abundance (% of sequences) are presented in Supplementary Table 2, with some differences observed in tubes with ABX− or ABX+ inoculum. Fusobacteriota and Proteobacteria were higher in tubes containing ABX− inoculum (*p* < 0.01), whereas the remaining phyla were not different. At the genus level, tubes containing the ABX− inoculum had higher (*p* < 0.05) abundances of *Bacteroides, [Ruminococcus]_gauvreauii_group, Enterococcus, Lactobacillus, Negativibacillus,* and *Fusobacterium*. In baseline (0 h) tubes containing ABX+ inoculum, *Adlercreutzia, [Ruminococcus]_gnavus_group, Anaeroplasma,* Lachnospiraceae unclassified, *Anaerobiospirillum*, *Peptococcus, Peptostreptococcus, UCG-005, Cetobacterium, Escherichia-Shigella, Morganella,* and *Proteus* were higher (*p* < 0.05).

### Pectin fermentation

**Figure 1:**
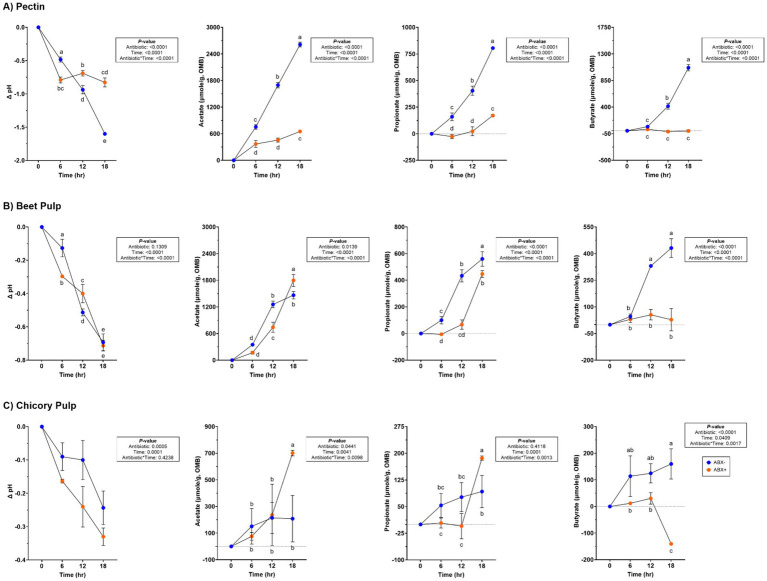
pH and SCFA concentrations (μmol/g, organic matter basis) of tubes containing **(A)** pectin, **(B)** beet pulp, and **(C)** chicory pulp. Data are presented as change from baseline (0 h) least square means ± SEM.

Lower pH values (*p* < 0.0001) were observed in tubes containing ABX− inoculum and increased concentrations of SCFA (*p* < 0.0001; Supplementary Table 3; Figure 1). Acetate concentrations increased (*p* < 0.0001) over time in tubes with ABX+ inoculum, whereas propionate concentrations only increased after 12 h (*p* < 0.0001). Butyrate concentrations remained unaltered throughout fermentation in ABX+ tubes.

**Figure 2:**
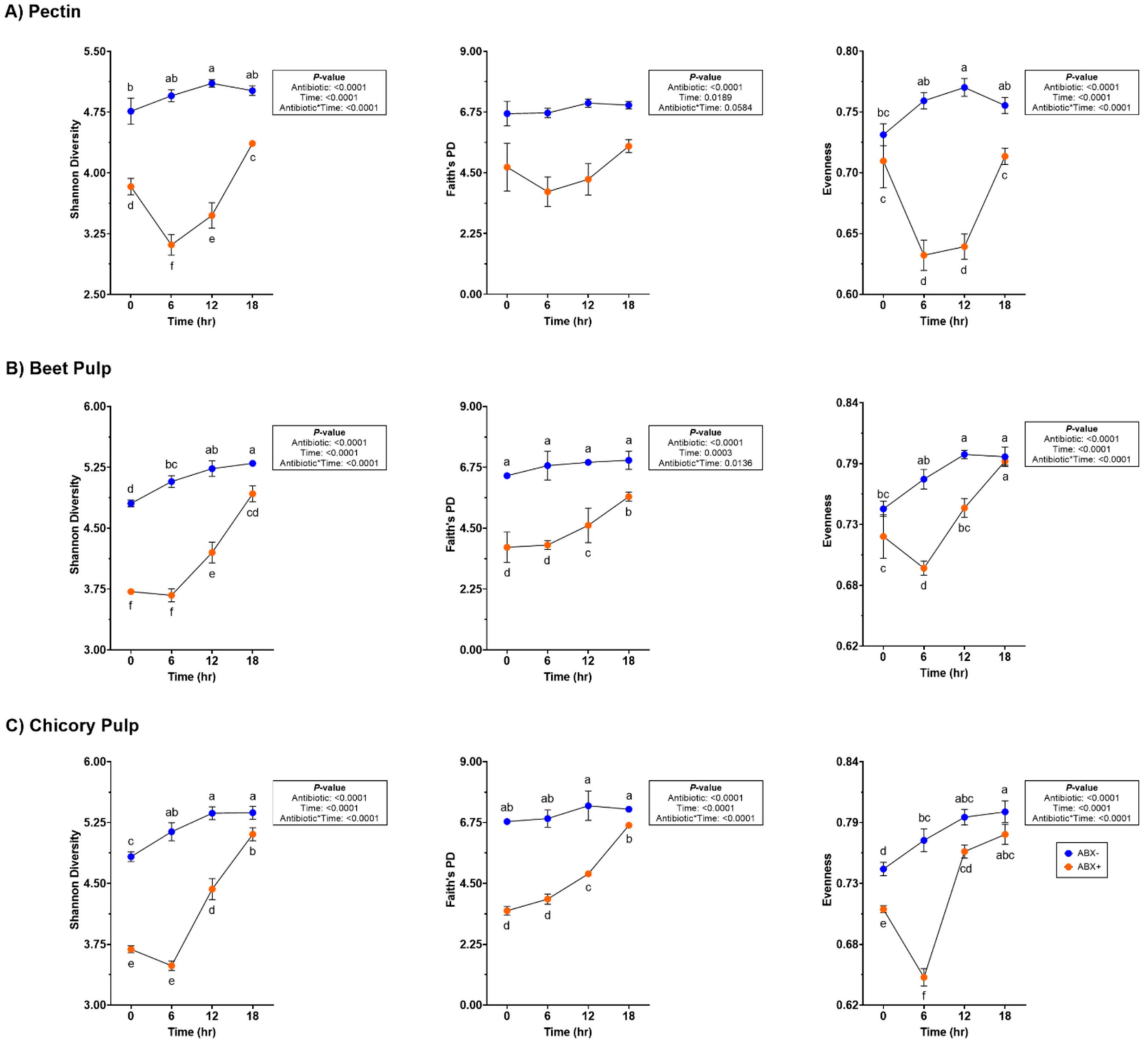
Bacterial alpha diversity measures of tubes containing **(A)** pectin, **(B)** beet pulp, and **(C)** chicory pulp. Data are presented as least square means ± SEM.

Fecal bacterial alpha diversity measures [Shannon Diversity Index, Faith’s Phylogenetic Diversity (PD)] were lower in tubes containing ABX+ inoculum (*p* < 0.0001; Supplementary Table 4; Figure 2). Evenness measures were similar at the start of fermentation but decreased in tubes containing ABX+ inoculum until 18 h, when measures returned to baseline (0 h) measures (*p* < 0.0001). None of the alpha diversity parameters were similar after 18 h. Principal coordinate analysis plots demonstrated shifts in the bacterial beta diversity of *in vitro* fermentation tubes containing pectin (Supplementary Figure 1). Both unweighted and weighted plots demonstrated separate clustering by metronidazole treatment during fermentation.

**Figure 3:**
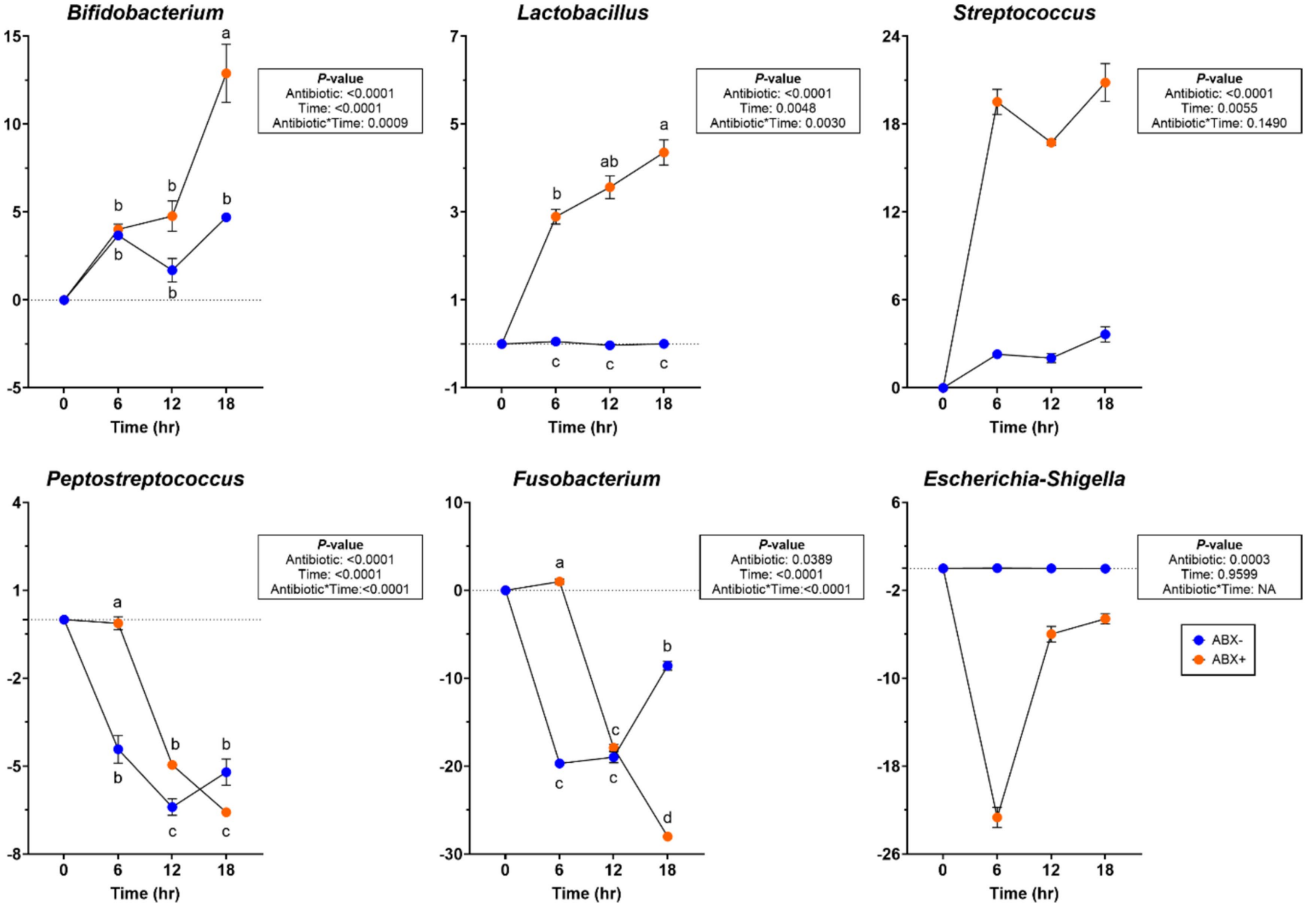
Bacterial genera relative abundances (% of sequences) during pectin *in vitro* fermentation using fecal inocula collected from dogs before (ABX−) and after (ABX+) metronidazole administration. Data are presented as change from baseline (0 h) least square means ± SEM.

At the bacterial phylum level, the relative abundances of Actinobacteria and Firmicutes increased (*p* < 0.05; Supplementary Table 5) in tubes containing ABX+ inoculum, whereas the relative abundance of Bacteriodota increased (*p* < 0.0001) in tubes containing ABX− inoculum. The relative abundance of Fusobacteriota decreased over time (*p* < 0.0001), and the relative abundance of Proteobacteria showed greater reduction in tubes with ABX+ inoculum (*p* < 0.0001). During pectin fermentation, many genera were affected by metronidazole treatment (Supplementary Table 5). Tubes containing ABX+ inoculum showed increased relative abundance of *Allobaculum* and *Enterococcus* after 18 h (*p* < 0.001). *Bacteroides, Blautia, and Peptoclostridium* relative abundances increased, whereas *Clostridium_sensu_stricto_1* relative abundance decreased (*p* < 0.0001) in tubes with ABX− inoculum. In tubes containing ABX+ inoculum, *Bifidobacterium, Lactobacillus,* and *Streptococcus* relative abundances increased more, whereas *Peptostreptococcus* and *Escherichia-Shigella* relative abundances decreased (*p* < 0.001; Figure 3). *Fusobacterium* relative abundance increased after 6 h in tubes containing ABX+ inoculum but decreased for the remainder of fermentation (*p* < 0.0001; Figure 3).

### Beet pulp fermentation

Variation in pH values was observed throughout fermentation, but overall, it declined over time (*p* < 0.0001; Supplementary Table 3; Figure 1). All SCFA concentrations increased with time, but propionate and butyrate concentrations were higher in tubes containing the ABX− inoculum by 18 h (*p* < 0.0001). Minor increases in butyrate concentration were observed throughout fermentation.

**Figure 4:**
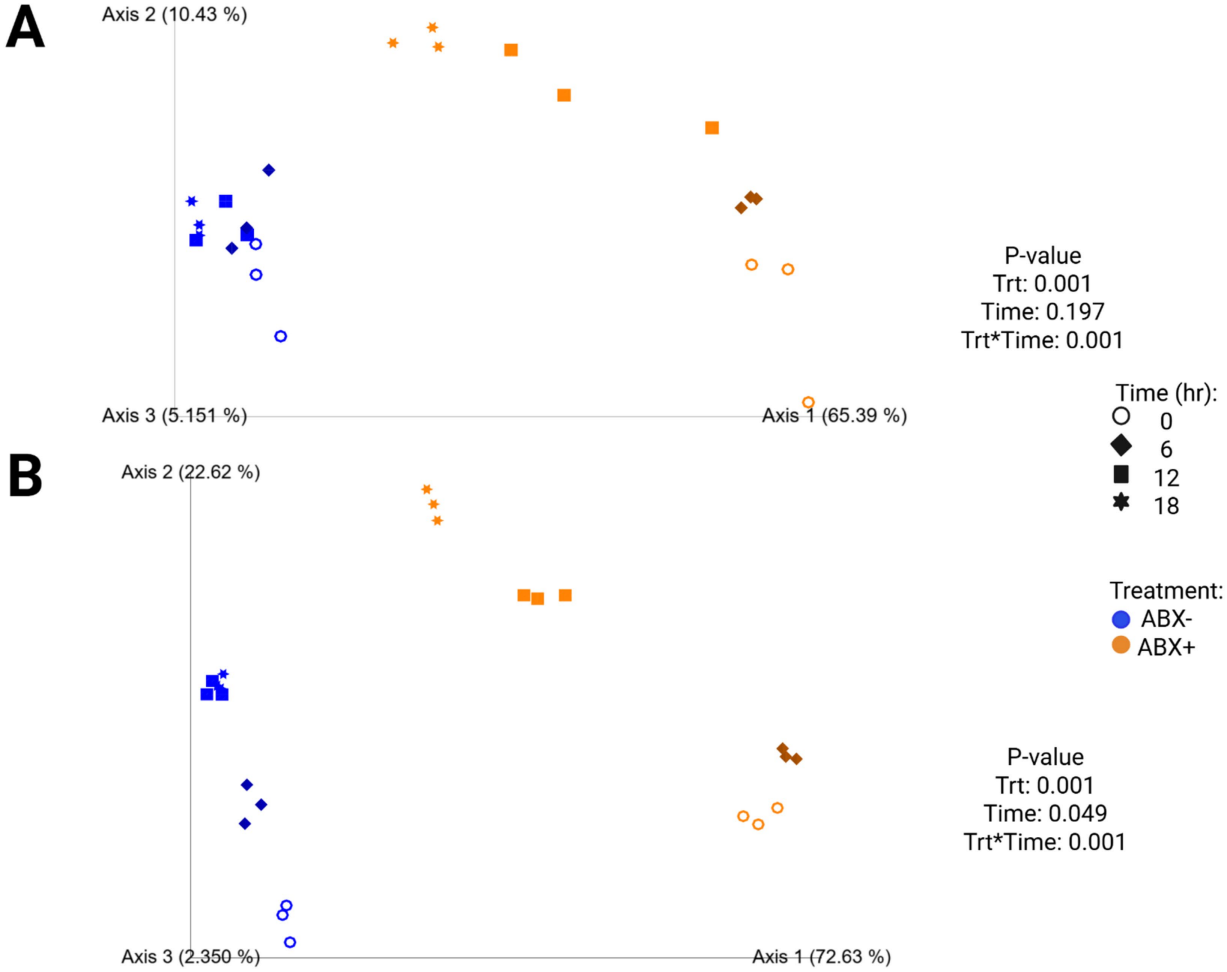
Unweighted **(A)** and weighted **(B)** principal coordinates analysis plots of *in vitro* fermentation tubes containing beet pulp.

Increased fecal bacterial alpha diversity was observed in tubes containing ABX− inoculum (*p* < 0.0001; Supplementary Table 4; Figure 2). Over time, the Shannon Diversity Index and Faith’s PD measures increased in tubes containing ABX+ inoculum but were lower than those of tubes with ABX− inoculum at the end of fermentation. Evenness decreased after 6 h in tubes containing ABX+ inoculum but increased to achieve similar measures as tubes containing ABX− inoculum after 18 h (*p* < 0.0001). Beta diversity plots demonstrating fecal bacterial shifts during fermentation are presented in Figure 4. The unweighted plot demonstrated significant clustering by antibiotic treatment (*p* = 0.001), with shifts of tubes containing ABX+ inoculum away from 0 h toward tubes containing ABX− inoculum. The weighted beta diversity plot demonstrates similar shifts with separation by antibiotic treatment (*p* = 0.001) and time (*p* = 0.049).

**Figure 5:**
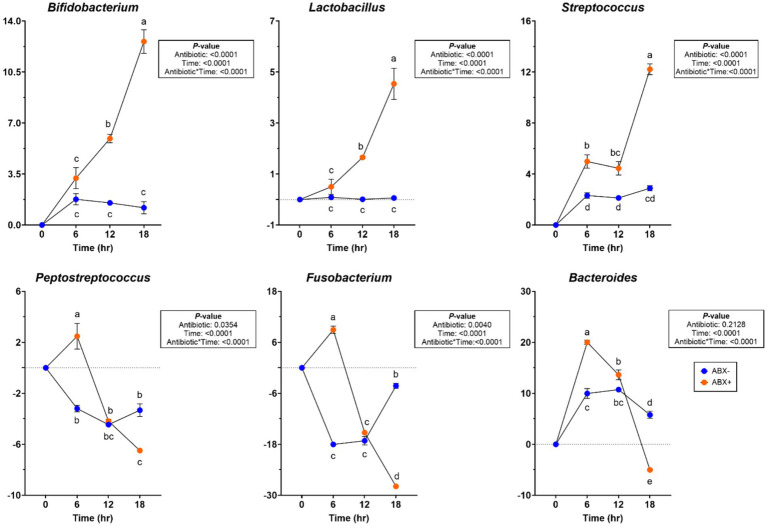
Bacterial genera relative abundances (% of sequences) during beet pulp *in vitro* fermentation using fecal inocula collected from dogs before (ABX−) and after (ABX+) metronidazole administration. Data are presented as change from baseline (0 h) least square means ± SEM.

At the bacterial phylum level, the relative abundances of Actinobacteridota and Bacteroidota increased (*p* < 0.01) over time (Supplementary Table 6). In tubes containing ABX+ inoculum, Firmicutes relative abundance was increased at 6 h in tubes containing ABX+ inoculum (*p* < 0.05), while Proteobacteria relative abundance had greater reductions (*p* < 0.001). At the bacterial genus level, *Blautia* and *Peptoclostridium* relative abundances increased more (*p* < 0.0001), while *Clostridium_sensu_stricto_1* and *Parasutterella* relative abundances had greater reductions in tubes containing ABX− inoculum (*p* < 0.001). Relative abundances of *Bifidobacterium*, *Lactobacillus,* and *Streptococcus* increased in tubes containing ABX+ inoculum after 18 h (*p* < 0.0001; Figure 5). Relative abundances of *Peptostreptococcus, Fusobacterium,* and *Bacteroides* increased after 6 h in tubes containing ABX+ inoculum and declined the most by 18 h (*p* < 0.0001; Figure 5).

### Chicory pulp fermentation

No significant antibiotic*time was observed for pH during chicory pulp fermentation, but tubes containing ABX+ inoculum showed larger pH reductions (*p* < 0.001; Supplementary Table 3; Figure 1). Acetate and propionate concentrations increased over time, with ABX+ inoculum demonstrating higher concentrations by 18 h (*p* < 0.01). Butyrate concentrations were higher in tubes with ABX− inoculum but were decreased in tubes with ABX+ inoculum by 18 h (*p* < 0.01).

**Figure 6:**
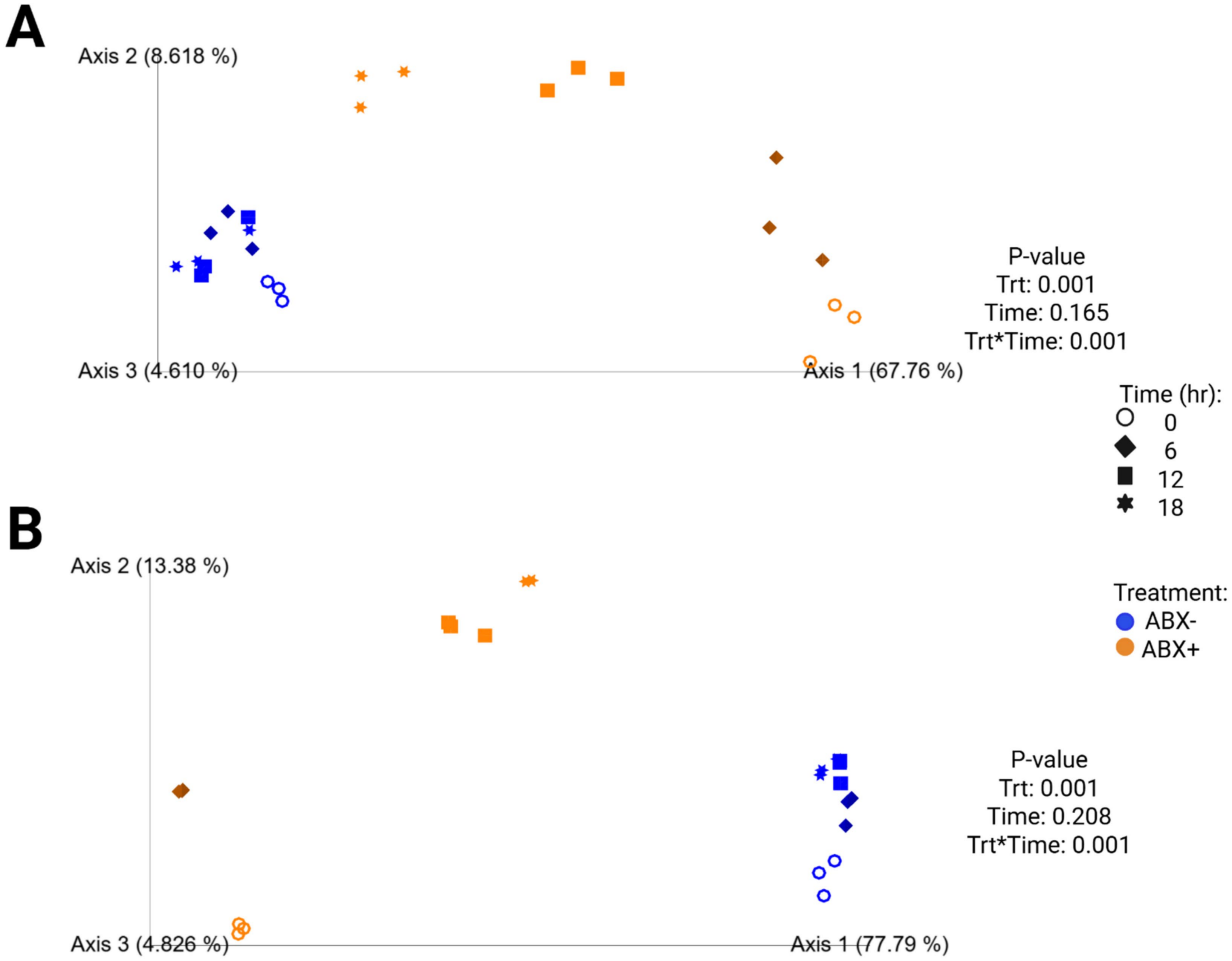
Unweighted **(A)** and weighted **(B)** principal coordinates analysis plots of *in vitro* fermentation tubes containing chicory pulp.

In tubes containing chicory pulp, the Shannon Diversity Index and evenness increased (*p* < 0.0001; Supplementary Table 4; Figure 2) in tubes containing ABX− inoculum, while Faith’s PD was not significantly different across 18 h. In tubes containing ABX+ inoculum, the Shannon Diversity Index and Faith’s PD increased over time, while evenness decreased after 6 h (*p* < 0.0001). After 18 h, tubes containing ABX+ inoculum showed increased evenness measures and were not different from tubes containing ABX− inoculum. Beta diversity plots demonstrating fecal bacterial shifts during fermentation of chicory pulp are presented in Figure 6. Significant clustering is demonstrated within antibiotic treatment (p=0.001) of both unweighted and weighted plots. The unweighted plot demonstrates shifts of tubes containing ABX+ inoculum away from 0 h toward tubes containing ABX- inoculum. In the weighted beta diversity plot, similar shifts were observed, with tubes containing ABX+ inoculum shifting towards tubes containing ABX- inoculum by 18 h.

**Figure 7:**
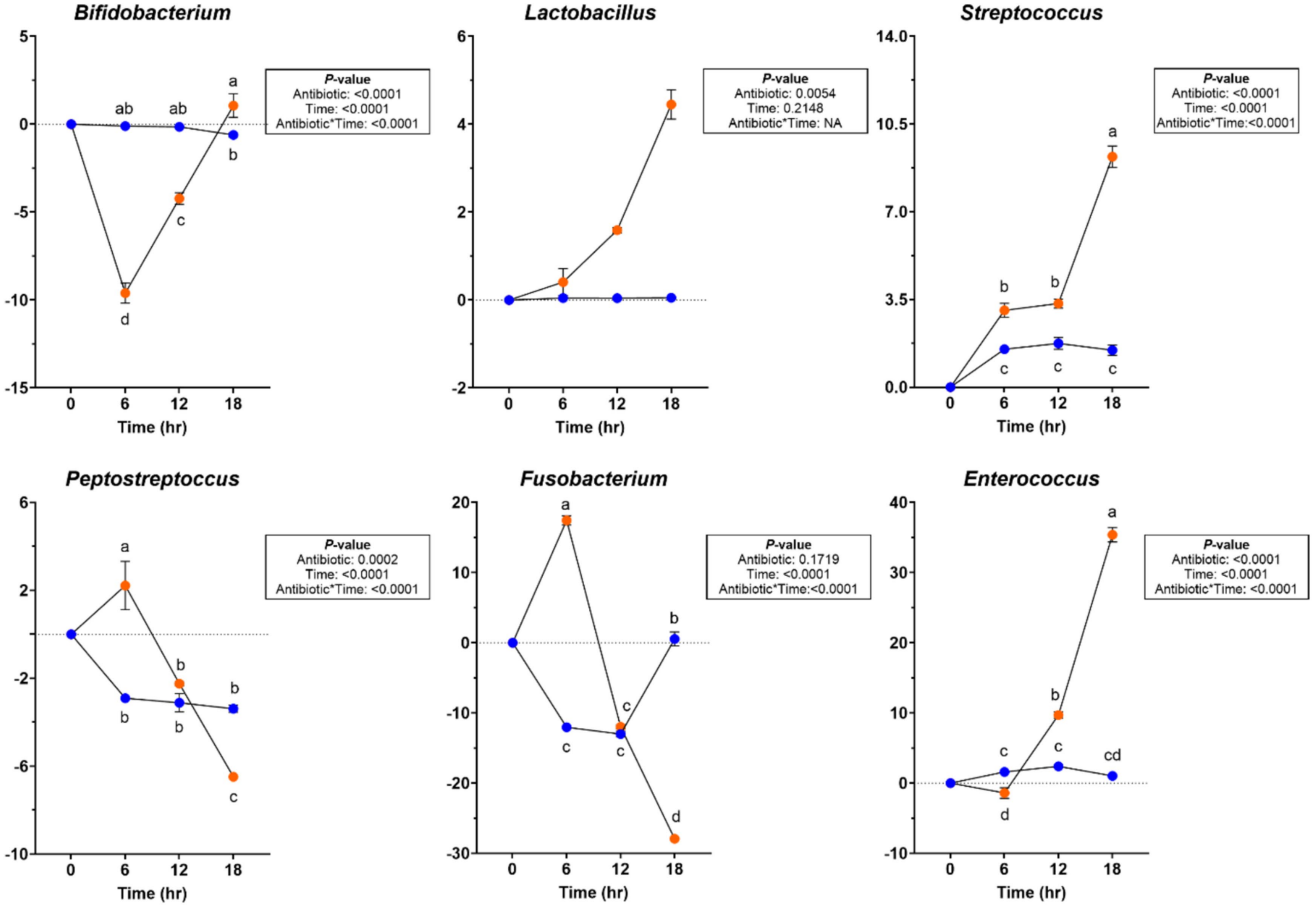
Bacterial genera relative abundances (% of sequences) during chicory pulp *in vitro* fermentation using fecal inocula collected from dogs before (ABX−) and after (ABX+) metronidazole administration. Data are presented as change from baseline (0 h) least square means ± SEM.

Significant changes were observed in the bacterial phyla and genera levels during chicory pulp fermentation (Supplementary Table 6). Actinobacteroidota relative abundance increased after 6 h in tubes containing ABX− inoculum but decreased across all other tubes for the remainder of fermentation (*p* < 0.0001). Firmicutes relative abundance increased (*p* < 0.0001), whereas Proteobacteria relative abundance showed a greater reduction in tubes containing ABX+ inoculum (*p* < 0.0001). Bacteroidota relative abundance increased and Fusobacteriota relative abundance decreased (*p* < 0.0001) throughout fermentation. At the bacterial genus level, the relative abundances of *Bacteroides* and *Peptoclostridium* increased until 12 h of fermentation but then decreased by 18 h in tubes containing ABX+ inoculum (*p* < 0.0001). *Bifidobacterium* and *Allobaculum* relative abundances decreased until 12 h but increased after 18 h (*p* < 0.0001), while *Peptostreptococcus* and *Fusobacterium* relative abundances increased after 6 h but had greater reductions at the end of fermentation (*p* < 0.0001; Figure 7). *Lactobacillus, Streptococcus,* and *Enterococcus* relative abundances increased more in tubes containing ABX+ inoculum (*p* < 0.01; Figure 7).

### Cellulose fermentation

A significant antibiotic*time effect was observed during cellulose fermentation but varied with time. The pH increased in tubes with ABX− inoculum and declined in tubes with ABX+ inoculum (*p* < 0.01; Supplementary Table 3), but after 18 h of fermentation, the pH was similar to that of the baseline (0 h) measures. Propionate concentrations were higher in tubes with ABX+ inoculum at 12 h (*p* < 0.05), but were not different at any other time point. Acetate and butyrate concentrations were not significantly affected by antibiotics or treatment duration.

The bacterial alpha diversity measures of cellulose fermentation are presented in Supplementary Table 4. Shannon Diversity Index and Faith’s PD measures in tubes containing ABX+ inoculum increased over time and were not different from those in tubes containing ABX− inoculum after 18 h (*p* < 0.0001). Evenness was similar at baseline (0 h) in tubes containing either ABX− or ABX+ inoculum and was not different at the end of fermentation (*p* < 0.0001). Supplementary Figure 2 presents the fecal bacterial shifts during cellulose fermentation. Unweighted and weighted plots demonstrate clustering by metronidazole treatment and within time points, with tubes containing ABX+ inoculum shifting toward tubes containing ABX− inoculum.

Changes in bacterial phyla and genera during fermentation are presented in Supplementary Table 8. No significant antibiotic*time effects were observed at the bacterial phyla level. *Bifidobacterium, Collinsella, Allobaculum, Dubosiella, Enterococcus*, Erysipelotrichaceae uncultured, *Lactobacillus, Streptococcus, Escherichia-Shigella,* and *Proteus* relative abundances increased in tubes containing ABX+ inoculum by 18 h (*p* < 0.05). Relative abundances of *Bacteroides, [Ruminococcus]_torques_group, Clostridium_sensu_stricto_1, Fusobacterium, Peptoclostridium, Peptostreptococcus,* and *Phascolarctobacterium* were increased in tubes containing ABX+ inoculum at 6 h but decreased by 18 h (*p* < 0.05). In tubes containing ABX+ inoculum, *[Eubacterium]_brachy_group, [Eubacterium]_nodatum_group, Alloprevotella,* Lachnospiraceae unclassified, Lachnospiraceae uncultured, *Negativibacillus, Oribacterium,* and *Peptococcus* relative abundances increased at 12 h but decreased (*p* < 0.0001) by 18 h.

## Discussion

Research continues to demonstrate that antibiotic usage can drastically and negatively alter microbial fermentation, activity, and composition; however, bactericidal antibiotics as a therapeutic strategy for GI enteropathies remain common in veterinary medicine (1, 2). In this study, metronidazole was selected, as several studies using dogs and cats have demonstrated potent effects within the GI microbiome, including the reduction of fecal bacterial diversity, microbial-derived metabolites, and loss of many commensal taxa (3, 5, 7–11, 13, 30, 31). Strategies to mitigate the consequences associated with antibiotic administration, such as dietary intervention or supplementation of functional ingredients, may assist in increasing commensal and SCFA-producing bacteria. While the focus of this discussion will be on the effects of metronidazole or dietary fiber, it should be noted that biotic usage (e.g., pre-, pro-, and post-biotics) has been shown to beneficially modulate the GI microbiome, enhance intestinal barrier function, and aid in the immune response, as summarized by Wilson and Swanson (32), and is also common in treatment plans for patients with a range of GI ailments or chronic enteropathies.

In healthy dogs, dietary fiber has been shown to increase counts of beneficial bacteria, including SCFA-producers (*Bacteroides, Prevotella, Faecalibacterium, Peptostreptococcus,* and *Allobaculum*) and lactic acid bacteria (LAB; *Bifidobacterium* and *Lactobacillus*), reduce counts of opportunistic pathogens such as *Fusobacterium* and *Streptococcus* (11, 14, 33–36), and increase SCFA concentrations in canine stool samples (37–41). Of the studies mentioned, beet pulp, chicory pulp, soybean hulls, inulin, and Miscanthus fiber were included as fiber sources in those diets. While the current study tested four fibers (cellulose, pectin, beet pulp, and chicory pulp), using a broader panel, including inulin, resistant starches, non-digestible oligosaccharides (e.g., arabinoxylans), and gums, might yield more comprehensive insights into fiber fermentation patterns and should be considered for future studies.

In the current experiment, several SCFA-producing bacteria and SCFA concentrations were increased in tubes with pre-metronidazole inoculum. As anticipated, metronidazole administration affected SCFA concentrations, with results demonstrating minimal or depleted butyrate concentrations. In the current study, beet pulp and chicory pulp yielded some butyrate, which was depleted after 12 h of fermentation. Butyrate is crucial to intestinal cells, as it enhances intestinal barrier integrity, is considered anti-inflammatory, is a cofactor in cellular functions (e.g., transcriptional activation and histone modification), and serves as the primary energy source for colonocytes (42). Considering its many roles, the lack of intestinal concentrations of butyrate and butyrate-producing bacteria is strongly associated with chronic GI inflammation (e.g., irritable bowel disease), which could lead to worsening of the disease and is being studied as a potential clinical therapy. Dietary strategies targeting butyrate production may include the addition of prebiotic fibers selectively utilized by butyrate-producing bacteria or increasing the total dietary fiber intake with greater fractions of fermentable fibers. Strategies such as these could promote butyrate production to restorative levels and avoid GI inflammation observed in low-butyrate conditions.

In veterinary medicine, cellulose has demonstrated improvements in fecal quality, increases in fecal bulk, and aids in the recovery of acute diarrhea (43–45), as it is minimally fermented and insoluble (17, 37). However, highly fermentable and soluble fibers, such as pectin, are rapidly fermented by microbes and were recently shown to have higher gas production compared with cellulose and acacia fiber (46), which could cause extensive flatulence and discomfort in animals, especially in those with concurrent GI distress. As this was an *in vitro* assay, flatulence was not an observable outcome. However, it was hypothesized that the increased consumption of fibers with higher fermentability may lead to increased gas production *in vivo*. Although gas production was not an outcome of this study, it is important to recognize that fiber sources can vary greatly, and special consideration should be given to animals with active GI issues to avoid additional GI stress.

There are a multitude of factors that can influence the GI tract, including the health status of the animal (e.g., healthy vs. chronic GI disease), drug usage (e.g., antibiotics), and diet. Any of these factors or a combination thereof can influence microbial composition and functions. Reductions in luminal pH can influence the abundance of pH-sensitive bacteria (e.g., lowering the abundance of *Streptococcus*) (47) in addition to disrupting microbial metabolism and SCFA production. In the current study, the pH of the fermentation media decreased significantly and showed greater pH reduction after 18 h of fermentation in pre-metronidazole inoculum during pectin fermentation, with no statistical differences observed by the end of beet pulp or chicory pulp fermentation. Previous *in vitro* fermentation studies testing highly fermentable fibers (i.e., pectin, inulin, and short-chain fructooligosaccharides) demonstrated greater pH reductions compared to nonfermentable cellulose (17, 46). Similar results were observed in the current study, as the pH significantly declined during the fermentation of moderate-to-high fermentable fibers. Metronidazole usage limited pH alterations during pectin fermentation, and fewer changes were observed during chicory pulp fermentation.

As previously stated, pH can influence the GI microbiota, and the abundance of LAB (e.g., *Bifidobacterium, Lactobacillus,* and *Streptococcus*) thrives under more acidic conditions, which may explain the results obtained during pectin, beet pulp, and chicory pulp fermentation with post-metronidazole inoculum. Although the changes during chicory pulp fermentation were to a lesser extent in comparison, previous studies have demonstrated similar microbial patterns in dogs (5, 7, 10) and cats (6) treated with metronidazole. Lactate is a byproduct of anaerobic bacteria, and in cases of rapid fermentation or overproduction, it can lower the pH and sustain the growth of other LAB (14, 48). In the current study, the abundance of LAB increased with post-metronidazole inoculum and continued to increase throughout fermentation. Previous research has demonstrated that increased and prolonged systemic concentrations of lactate can have neurotoxic effects on the host (49). Translating this information to *in vivo* is challenging, as concentrations were not quantified in the present study, but given the rapid and significant increase in LAB during fermentation, we hypothesized that lactate concentrations would have also increased. Measurement of lactate production would be of interest in future *in vitro* models of antibiotic-treated or clinically ill patients.

Using an *in vitro* fermentation system as a model for mimicking the GI system of animals is not without its limitations and should be addressed in the present study. First, the *in vitro* fermentation assay is a closed system that is temperature-controlled and anaerobically maintained but does not correct for host GI secretions (e.g., bile acids, mucus, and enzymes), as observed *in vivo*. Research has shown significant correlations between bacterial-derived metabolites (e.g., SCFA, bile acids, and uremic toxins from bacterial protein metabolism) and widespread systemic effects within the host (e.g., brain, liver, and kidney). Considering how these metabolites could travel throughout the body, the buildup of these products within the *in vitro* system could have additional effects on microbial composition and activity, such as utilization in microbial cross-feeding (e.g., lactate metabolism), which would otherwise be lacking with *in vivo* models. Additionally, the host GI tract is highly variable (e.g., oxygen concentration and luminal pH) and not sufficiently replicated in an *in vitro* model. Physiological properties of a host GI tract can influence the microbiota and its respective functions, metabolism, and interaction with other commensal bacteria, thereby affecting cross-feeding and downstream secondary microbial metabolism. In addition, the microbiological medium utilized in this assay is formulated to provide essential nutrients that microbes need to survive the transition from host to *in vitro* closed systems; however, this may influence the microbial activity and contribute to a lag phase of growth. In this assay, only four time points (0, 6, 12, and 18 h) were sampled to measure outcomes; however, more frequent sampling could have provided a more in-depth explanation of fermentation kinetics and potential insight into lag phase functions. The four test fibers selected are common to the pet food industry but represent a small fraction of what may be used in commercial diets. Expanding the panel of fibers tested with more diverse characteristics (e.g., fermentability and solubility) may have demonstrated additional novel outcomes for application in personalized or targeted pet food formulations or supplemental products targeted toward beneficial microbial shifts and metabolic outcomes. Finally, translating these data to the general canine population may be limited, as only four dogs were selected for this study and of similar backgrounds; therefore, using a more diverse population may have provided different outcomes.

Another limitation that should be addressed is related to the animals selected for inoculum collection. All four donors were of the same breed, of similar age, and kept in identical environmental conditions. While these dogs are not genetically related, applying this information to other breeds and health conditions is somewhat limited because breed, age, activity status, and diet can affect the microbiome (11). This study was performed under tightly controlled conditions with a strict population; however, future studies should consider testing these variables to evaluate the similarity in patterns. In summary, the *in vitro* assay can be beneficial for testing substrates of interest; however, this method does not allow testing of the full variety of dietary fibers within the pet food industry, permit the removal of bacterial byproducts/waste, or allow for mimicking host-microbe metabolism and absorption patterns, as observed *in vivo*. The results generated from this analysis have limited *in vivo* translation but have provided beneficial insights into fiber fermentation patterns following potent antibiotic treatment that may assist dietary recommendations during recovery.

In conclusion, metronidazole had significant negative effects on the GI microbiome, as demonstrated by the reduction in microbial abundance, bacterial diversity, and bacterial fermentative metabolites. Using an *in vitro* fermentation system provided with fibers of varying properties (e.g., fermentative potential and solubility) demonstrated reductions in pH, increased SCFA concentrations, alterations to alpha diversity measures, and beneficial shifts to beta diversity in tubes with pre-metronidazole inoculum. Significant increases in lactic acid bacteria (e.g., *Bifidobacterium, Lactobacillus*) and *Streptococcus* abundances were observed in tubes with post-metronidazole inoculum during beet pulp and pectin fermentation, indicating that these bacteria might be more opportunistic, whereas SCFA-producers (e.g., *Bacteroides, Blautia, Faecalibacterium, Fusobacterium*) decreased. Butyrate production was limited in all tubes with post-metronidazole inocula, but increased concentrations of acetate and propionate were observed during beet pulp and chicory pulp fermentation, and to a lesser extent, during pectin fermentation. From these results, we can conclude that while antibiotic administration may be essential for treatment in practice, special consideration should be given to its effects beyond its intended purpose, such as the effects observed with secondary microbial metabolism, especially with regard to dietary fiber fermentation patterns.

## Data Availability

The original contributions presented in the study are included in the article/Supplementary material, further inquiries can be directed to the corresponding author. All sequence data used for analysis are available at the NCBI sequence read archive under BioProject PRJNA1268972 (https://www.ncbi.nlm.nih.gov/bioproject/PRJNA1268972).

## References

[1] Weese JS Taylor-Rakocevic ME Topdjian K Battersby I. Antimicrobial dispensing for common conditions in dogs and cats at a large veterinary practice network, 2023. Vet J. (2025) 312:106374. doi: 10.1016/j.tvjl.2025.106374, PMID: 40383355

[2] Robbins SN Goggs R Kraus-Malett S Goodman L. Effect of institutional antimicrobial stewardship guidelines on prescription of critically important antimicrobials for dogs and cats. J Vet Intern Med. (2024) 38:1706–17. doi: 10.1111/jvim.17043, PMID: 38465850 PMC11099728

[3] Fenimore A Martin L Lappin MR. Evaluation of metronidazole with and without *Enterococcus faecium* SF68 in shelter dogs with diarrhea. Top Companion Anim Med. (2017) 32:100–3. doi: 10.1053/j.tcam.2017.11.001, PMID: 29291770

[4] Singleton DA Noble PJM Sánchez-Vizcaíno F Dawson S Pinchbeck GL Williams NJ. Pharmaceutical prescription in canine acute diarrhoea: a longitudinal electronic health record analysis of first opinion veterinary practices. Front Vet Sci. (2019) 6:218. doi: 10.3389/fvets.2019.00218 31334254 PMC6615257

[5] Belchik SE Oba PM Lin C-Y Swanson KS. Effects of a veterinary gastrointestinal low-fat diet on fecal characteristics, metabolites, and microbiota concentrations of adult dogs treated with metronidazole. J Anim Sci. (2024) 102:skae297. doi: 10.1093/jas/skae297 39344678 PMC11568346

[6] Belchik SE Oba PM Lin C-Y Swanson KS. Effects of a veterinary gastrointestinal diet on fecal characteristics, metabolites, and microbiota concentrations of adult cats treated with metronidazole. J Anim Sci. (2024) 102:skae274. doi: 10.1093/jas/skae274, PMID: 39279199 PMC11465373

[7] Belchik SE Oba PM Wyss R Asare PT Vidal S Miao Y. Effects of a milk oligosaccharide biosimilar on fecal characteristics, microbiota, and bile acid, calprotectin, and immunoglobulin concentrations of healthy adult dogs treated with metronidazole. J Anim Sci. (2023) 101:skad011. doi: 10.1093/jas/skad011, PMID: 36617268 PMC9912710

[8] Chaitman J Ziese A Pilla R Minamoto Y Blake AB Guard BC. Fecal microbial and metabolic profiles in dogs receiving either fecal microbiota transplantation or oral metronidazole. Front Vet Sci. (2020) 7:192. doi: 10.3389/fvets.2020.00192 32363202 PMC7182012

[9] Marshall-Jones ZV Patel KV Castillo-Fernandez J Lonsdale ZN Haydock R Staunton R. Conserved signatures of the canine faecal microbiome are associated with metronidazole treatment and recovery. Sci Rep. (2024) 14:5277. doi: 10.1038/s41598-024-51338-7, PMID: 38438389 PMC10912219

[10] Pilla R Gaschen FP Barr JW Olson E Honneffer J Guard GC. Effects of metronidazole on the fecal microbiome and metabolome in healthy dogs. J Vet Intern Med. (2020) 34:1853–66. doi: 10.1111/jvim.15871 32856349 PMC7517498

[11] Shah H Trivedi M Gurjar T Sahoo DK Jergens AE Yadav VK. Decoding the gut microbiome in companion animals: impacts and innovations. Microorganisms. (2024) 12:1831. doi: 10.3390/microorganisms12091831, PMID: 39338505 PMC11433972

[12] Guard BC Barr JW Lavanya R Klemashevich C Jayaraman A Steiner JM. Characterization of microbial dysbiosis and metabolomics changes in dogs with acute diarrhea. PLoS One. (2015) 10:e0127259. doi: 10.1371/journal.pone.0127259 26000959 PMC4441376

[13] Martini SE Schmidt T Huang W Blake AB Cavasin JP Suchodolski JS. Effects of metronidazole on the fecal microbiota, fecal metabolites, and serum metabolites of healthy adult cats. Pets. (2025) 2:19. doi: 10.3390/pets2020019

[14] Pilla R Suchodolski JS. The role of the canine gut microbiome and metabolome in health and gastrointestinal disease. Front Vet Sci. (2020) 6:498. doi: 10.3389/fvets.2019.00498, PMID: 31993446 PMC6971114

[15] Mishra BP Mishra J Paital B Ruth PK Jena MK Reddy BVV. Properties and physiological effects of dietary fiber-enriched meat products: a review. Front Nutr. (2023) 10:1275341. doi: 10.3389/fnut.2023.1275341 38099188 PMC10720595

[16] Moreno AA Parker VJ Winston JA Rudinsky AJ. Dietary fiber aids in the management of canine and feline gastrointestinal disease. J Am Vet Med Assoc. (2022) 260:S33–45. doi: 10.2460/javma.22.08.0351, PMID: 36288203

[17] de Godoy MRC Mitsuhashi Y Bauer LL Fahey GC Buff PR Swanson KS. In vitro fermentation characteristics of novel fibers, coconut endosperm fiber and chicory pulp, using canine fecal inoculum. J Anim Sci. (2015) 93:370–6. doi: 10.2527/jas.2014-7962, PMID: 25403197

[18] Fahey GC Merchen NR Corbin JE Hamilton AK Serbe KA Lewise SM. Dietary fiber for dogs: I. Effects of beet pulp on nutrient intake, digestibility, metabolizable energy, and digesta mean retention time. J Anim Sci. (1990) 68:4221–8. doi: 10.2527/1990.68124221x 1962765

[19] Association of American Feed Control Officials (AAFCO). Official publication 2023. Oxford (IN): AAFCO (2023).

[20] Laflamme DP. Development and validation of a body condition score system for dogs: a clinical tool. Canine Pract. (1997) 22:10–5.

[21] Cammarota G Ianiro G Tilg H Rajilić-Stojanović M Kump P Satokari R. European consensus conference on faecal microbiota transplantation in clinical practice. Gut. (2017) 66:569–80. doi: 10.1136/gutjnl-2016-313017, PMID: 28087657 PMC5529972

[22] Bourquin LD Titgemeyer EC Fahey GC. Vegetable fiber fermentation by human fecal bacteria: cell wall polysaccharide disappearance and short-chain fatty acid production during in vitro fermentation and water-holding capacity of unfermented residues. J Nutr. (1993) 123:860–9. doi: 10.1093/jn/123.5.860, PMID: 8387579

[23] Erwin ES Marco GJ Emery EM. Volatile fatty acid analyses of blood and rumen fluid by gas chromatography. J Dairy Sci. (1961) 44:1768–71. doi: 10.3168/jds.S0022-0302(61)89956-6

[24] Caporaso JG Lauber CL Walters WA Berg-Lyons D Huntley J Fierer N. Ultra-high throughput microbial community analysis on the Illumina HiSeq and MiSeq platforms. ISME J. (2012) 6:1621–4. doi: 10.1038/ismej.2012.8, PMID: 22402401 PMC3400413

[25] Callahan BJ McMurdie PJ Rosen MJ Han AW Johnson AJ Holmes SP. DADA2: high-resolution sample inference from Illumina amplicon data. Nat Methods. (2016) 13:581–3. doi: 10.1038/nmeth.3869, PMID: 27214047 PMC4927377

[26] Bokulich NA Kaehler BD Rideout JR Dillon M Bolyen E Knight R. Optimizing taxonomic classification of marker-gene amplicon sequences with QIIME 2's q2-feature-classifier plugin. Microbiome. (2018) 6:90. doi: 10.1186/s40168-018-0470-z, PMID: 29773078 PMC5956843

[27] Quast C Pruesse E Yilmaz P Gerken J Schweer T Yarza P. The SILVA ribosomal RNA gene database project: improved data processing and web-based tools. Nucleic Acids Res. (2013) 41:590–6. doi: 10.1093/nar/gks1219 PMC3531112 23193283

[28] Robeson MS O'Rourke DR Kaehler BD Ziemski M Dillon MR Foster JT. Rescript: reproducible sequence taxonomy reference database management. PLoS Comput Biol. (2021) 17:e1009581. doi: 10.1371/journal.pcbi.1009581, PMID: 34748542 PMC8601625

[29] Lozupone C Knight R. UniFrac: a new phylogenetic method for comparing microbial communities. Appl Environ Microbiol. (2005) 71:8228–35. doi: 10.1128/AEM.71.12.8228-8235.2005, PMID: 16332807 PMC1317376

[30] Igarashi H Maeda S Ohno K Horigome A Odamaki T Tsujimoto H. Effect of oral metronidazole or prednisolone on fecal microbiota in dogs. PLoS One. (2014) 9:e107909. doi: 10.1371/journal.pone.0107909 25229475 PMC4168260

[31] Langlois DK Koenigshof AM Mani R. Metronidazole treatment of acute diarrhea in dogs: a randomized double blinded placebo-controlled clinical trial. J Vet Intern Med. (2019) 34:98–104. doi: 10.1111/jvim.15664, PMID: 31742807 PMC6979100

[32] Wilson SM Swanson KS. The influence of “biotics” on the gut microbiome of dogs and cats. Vet Rec. (2024) 195:2–12. doi: 10.1002/vetr.4914, PMID: 39545542

[33] Garcia-Mazcorro JF Mills DA Murphy K Noratto G. Effect of barley supplementation on the fecal microbiota, caecal biochemistry, and key biomarkers of obesity and inflammation in obese db/db mice. Eur J Nutr. (2018) 57:2513–28. doi: 10.1007/s00394-017-1523-y, PMID: 28815303

[34] Panasevich MR Kerr KR Dilger RN Fahey GC Jr Guérin-Deremaux L Lynch GL. Modulation of the faecal microbiome of healthy adult dogs by inclusion of potato fibre in the diet. Brit J Nutr. (2015) 113:125–33. doi: 10.1017/S0007114514003274 25418803

[35] Phungviwatnikul T Lee AH Belchik SE Suchodolski JS Swanson KS. Weight loss and high-protein, high-fiber diet consumption impact blood metabolite profiles, body composition, voluntary physical activity, fecal microbiota, and fecal metabolites of adult dogs. J Anim Sci. (2022) 100:1–17. doi: 10.1093/jas/skab379 PMC8846339 34967874

[36] Phungviwatnikul T Alexander C Do S He F Suchodolski JS de Godoy MRC. Effects of dietary macronutrient profile on apparent total tract macronutrient digestibility and fecal microbiota, fermentative metabolites, and bile acids of female dogs after spay surgery. J Anim Sci. (2021) 99:skab225. doi: 10.1093/jas/skab225, PMID: 34333604 PMC8418634

[37] de Godoy MRC Kerr KR Fahey GC. Alternative dietary fiber sources in companion animal nutrition. Nutrients. (2013) 5:3099–117. doi: 10.3390/nu5083099 23925042 PMC3775244

[38] De Souza Nogueira JP He F Mangian HF Oba PM de Godoy MRC. Dietary supplementation of a fiber-prebiotic and saccharin-eugenol blend in extruded diets fed to dogs. J Anim Sci. (2019) 97:4519–31. doi: 10.1093/jas/skz293, PMID: 31634399 PMC6827403

[39] Detweiler KB He F Mangian HF Davenport GM de Godoy MRC. Effects of high inclusion of soybean hulls on apparent total tract macronutrient digestibility, fecal quality, and fecal fermentative end-product concentrations in extruded diets of adult dogs. J Anim Sci. (2019) 97:1027–35. doi: 10.1093/jas/skz015, PMID: 30649345 PMC6396230

[40] Finet S He F Clark LV de Godoy MRC. Functional properties of miscanthus fiber and prebiotic blends in extruded canine diets. J Anim Sci. (2022) 100:skac078. doi: 10.1093/jas/skac078 35279717 PMC9047183

[41] Nybroe S Horsman PB Krag K Hosbjerg TG Stenberg K Khakimov B. Alterations in healthy adult canine faecal microbiome and selected metabolites as a result of feeding a commercial complete synbiotic diet with *Enterococcus faecium* NCIMB 10415. Animals (Basel). (2022) 13:144. doi: 10.3390/ani13010144 36611752 PMC9817848

[42] Salvi PS Cowles RA. Butyrate and the intestinal epithelium: modulation of proliferation and inflammation in homeostasis and disease. Cells. (2021) 10:1775. doi: 10.3390/cells10071775, PMID: 34359944 PMC8304699

[43] Brigitta W Simone S Michaela H Britta D Ellen K. Influence of different cellulose types on feces quality of dogs. J Nutr. (2002) 132:1728S–9S. doi: 10.1093/jn/132.6.1728S 12042508

[44] Holz M Fritz J Suchodolski JS Werner M Unterer S. Effects of dietary cellulose on clinical and gut microbiota recovery in dogs with uncomplicated acute diarrhea: a randomized prospective clinical trial. J Am Vet Med Assoc. (2024) 263:169–77. doi: 10.2460/javma.24.07.0476 39536440

[45] Prola L Dobenecker B Mussa PP Kienzle E. Influence of cellulose fibre length on faecal quality, mineral excretion and nutrient digestibility in cat. J Anim Physiol Anim Nutr (Berl). (2010) 94:362–7. doi: 10.1111/j.1439-0396.2008.00916.x, PMID: 19663982

[46] De La Guardia-Hidrogo VM Geary EL Wilson SM Bauer LL Menton JF Vinay E. In vitro fermentation characteristics of acacia fiber using canine fecal inoculum. J Anim Sci. (2025) 103:skaf152. doi: 10.1093/jas/skaf152, PMID: 40326307 PMC12147028

[47] Ilhan ZE Marcus AK Kang D-W Rittmann BE Krajmalnik-Brown R. Ph-mediated microbial and metabolic interactions in fecal enrichment cultures. mSphere. (2017) 2:e00047-17. doi: 10.1128/mSphere.00047-17, PMID: 28497116 PMC5415631

[48] Blake AB Guard BC Honneffer JB Lidbury JA Steiner JM Suchodolski JS. Altered microbiota, fecal lactate, and fecal bile acids in dogs with gastrointestinal disease. PLoS One. (2019) 14:e0224454. doi: 10.1371/journal.pone.0224454, PMID: 31671166 PMC6822739

[49] Louis P Duncan SH Sheridan PO Walker AW Flint HJ. Microbial lactate utilisation and the stability of the gut microbiome. Gut Microbiome (Camb). (2022) 3:e3. doi: 10.1017/gmb.2022.3, PMID: 39295779 PMC11406415

