# Supplementary material for: In vitro fermentation characteristics of dietary fibers using fecal inocula from dogs fed a canned diet and treated with metronidazole
Source: Front Vet Sci. 2025 Oct 29;12:1670624. doi: 10.3389/fvets.2025.1670624 (PMC12604977; doi:10.3389/fvets.2025.1670624)
Supplement: Supplementary file 1 [file Data_Sheet_1.docx]

# **Supplementary Table 1.** Baseline (0 hr) pH and SCFA concentrations (μmole/g, OMB) of tubes containing pectin, beet pulp, chicory pulp, or cellulose

| Fiber |  | ABX-^1^ | ABX+ | SEM^2^ | p-value |
| --- | --- | --- | --- | --- | --- |
| Pectin | pH | 6.97 | 6.91 | 0.035 | 0.3185 |
|  | Acetate | 49.39 | 53.82 | 5.965 | 0.4859 |
|  | Propionate | 11.69 | 8.66 | 2.912 | 0.5023 |
|  | Butyrate | 9.33^a^ | 1.37^b^ | 1.183 | 0.0109 |
| Beet pulp | pH | 6.96 | 6.94 | 0.013 | 0.3486 |
|  | Acetate | 140.24^a^ | 113.51^b^ | 6.541 | 0.0343 |
|  | Propionate | 11.07 | 6.76 | 1.849 | 0.2387 |
|  | Butyrate | 5.20^a^ | -1.14^b^ | 1.194 | 0.0084 |
| Chicory pulp | pH | 6.97 | 6.97 | 0.010 | 0.8075 |
|  | Acetate | 51.90^a^ | 36.17^b^ | 3.463 | 0.0325 |
|  | Propionate | 8.81 | 2.33 | 1.836 | 0.0551 |
|  | Butyrate | 10.42 | 1.24 | 1.830 | 0.0630 |
| Cellulose | pH | 7.03 | 7.02 | 0.006 | 0.4918 |
|  | Acetate | 23.31 | 17.52 | 4.903 | 0.3807 |
|  | Propionate | 5.90 | 3.21 | 3.177 | 0.5405 |
|  | Butyrate | 5.53 | -0.92 | 1.383 | 0.0770 |

^1^ABX- = samples collected from dogs prior to antibiotic administration; ABX+ = samples collected after receiving metronidazole (20 mg/kg body weight twice daily) for two wk.
^2^SEM = pooled standard errors of the means.
^ab^Mean values within a row with unlike superscript letters differ using parametric analysis (P<0.05).

# **Supplementary Table 2.** Blank-corrected bacterial phyla and genera relative abundances (% of sequences) at baseline (0 hr) of tubes containing ABX- or ABX+ inoculum

| Phyla | Genera | ABX-^1^ | ABX+ | SEM^2^ | p-value |
| --- | --- | --- | --- | --- | --- |
| Actinobacteridota | | 0.25 | 2.27 | 0.725 | 0.0735 |
|  | *Adlercreutzia* | -0.02^b^ | 0.00^a^ | 0.007 | 0.0142 |
|  | *Bifidobacterium* | 0.35 | 2.28 | 0.730 | 0.0735 |
|  | *Collinsella* | -0.08 | -0.01 | 0.188 | 0.7479 |
|  | *Coriobacteriaceae_UCG-002* | 0.00 | 0.00 | 0.035 | 0.8915 |
|  | *Slackia* | -0.01 | 0.00 | 0.009 | 0.6407 |
| Bacteroidota | | 0.44 | 0.05 | 0.240 | 0.4185 |
|  | *Alloprevotella* | 0.09 | 0.03 | 0.026 | 0.0775 |
|  | *Bacteroides* | 0.20^z^ | 0.02^y^ | 0.052 | 0.0130 |
|  | *Muribaculaceae* | 0.15 | 0.01 | 0.181 | 1.0000 |
|  | *Parabacteroides* | 0.01 | 0.00 | 0.006 | 0.4591 |
|  | *Prevotella* | 0.01 | 0.00 | 0.007 | 0.9520 |
|  | *Prevotellaceae_Ga6A1_group* | -0.03 | 0.00 | 0.010 | 0.1387 |
|  | *Rikenellaceae_RC9_gut_group* | 0.00 | 0.00 | 0.004 | 0.4527 |
| Firmicutes | | -3.84 | -5.37 | 0.797 | 0.1895 |
|  | *[Eubacterium]_brachy_group* | -0.16 | 0.00 | 0.094 | 0.1387 |
|  | *[Eubacterium]_nodatum_group* | -0.03 | 0.00 | 0.019 | 0.4591 |
|  | *[Ruminococcus]_gauvreauii_group* | 0.02^z^ | 0.00^y^ | 0.010 | 0.0263 |
|  | *[Ruminococcus]_gnavus_group* | -0.17^y^ | -0.05^z^ | 0.045 | 0.0242 |
|  | *[Ruminococcus]_torques_group* | -0.07 | 0.00 | 0.029 | 0.1387 |
|  | *Allobaculum* | -1.66 | -0.84 | 0.035 | 0.1030 |
|  | *Anaeroplasma* | 0.01^b^ | 0.35^a^ | 0.065 | 0.0012 |
|  | *Blautia* | -0.07 | 0.02 | 0.038 | 0.0822 |
|  | *Butyricicoccus* | 0.00 | 0.00 | 0.007 | 0.9322 |
|  | *Clostridium_sensu_stricto_1* | 0.15 | -0.01 | 0.101 | 0.4470 |
|  | *Dubosiella* | 0.13 | 0.15 | 0.084 | 0.8876 |
|  | *Enterococcus* | -0.04^z^ | -3.80^y^ | 0.521 | 0.0056 |
|  | Erysipelotrichaceae uncultured | -0.02 | 0.00 | 0.254 | 0.6861 |
|  | *Faecalibacterium* | 0.01 | 0.01 | 0.008 | 0.5094 |
|  | *Faecalibaculum* | 0.03 | 0.01 | 0.027 | 0.4950 |
|  | *Lachnoclostridium* | 0.03 | 0.00 | 0.011 | 0.0706 |
|  | Lachnospiraceae unclassified | -0.03^y^ | 0.00^z^ | 0.014 | 0.0263 |
|  | Lachnospiraceae uncultured | -0.01 | 0.00 | 0.013 | 0.4590 |
|  | *Lactobacillus* | -0.01^z^ | -0.77^y^ | 0.131 | 0.0019 |
|  | *Megamonas* | 0.01 | 0.00 | 0.009 | 0.1386 |
|  | *Negativibacillus* | 0.06^a^ | 0.00^b^ | 0.014 | 0.0002 |
|  | *Oribacterium* | 0.00 | 0.00 | 0.007 | 0.7538 |
|  | *Peptoclostridium* | -1.27 | 0.02 | 0.281 | 0.0934 |
|  | *Peptococcus* | -0.07^y^ | 0.00^z^ | 0.013 | 0.0263 |
|  | *Peptostreptococcus* | -0.53^y^ | 0.01^z^ | 0.144 | 0.0004 |
|  | *Phascolarctobacterium* | 0.01 | 0.00 | 0.019 | 0.4591 |
|  | *Romboutsia* | 0.00 | 0.00 | 0.033 | 0.1387 |
|  | *Sellimonas* | 0.00 | 0.00 | 0.012 | 1.0000 |
|  | *Streptococcus* | -0.02 | -0.21 | 0.100 | 0.1870 |
|  | *Terrisporobacter* | 0.00 | -0.02 | 0.073 | 0.1387 |
|  | *Turicibacter* | 0.00 | 0.01 | 0.023 | 0.4509 |
|  | UCG-005 | -0.03^b^ | 0.00^a^ | 0.016 | 0.0181 |
| Fusobacteriota | | 2.93^z^ | 0.03^y^ | 0.384 | 0.0005 |
|  | *Cetobacterium* | -0.41^y^ | 0.01^z^ | 0.045 | 0.0002 |
|  | *Fusobacterium* | 3.33^z^ | 0.02^y^ | 0.389 | 0.0005 |
| Proteobacteria | | 0.23^y^ | 3.01^z^ | 0.320 | 0.0005 |
|  | *Anaerobiospirillum* | -0.01^b^ | 0.05^a^ | 0.014 | 0.0057 |
|  | *Escherichia-Shigella* | -0.01^y^ | 2.10^z^ | 0.282 | 0.0005 |
|  | *Morganella* | 0.00^y^ | 0.06^z^ | 0.006 | 0.0002 |
|  | *Parasutterella* | 0.17 | 0.22 | 0.074 | 0.6256 |
|  | *Proteus* | 0.00^b^ | 0.06^a^ | 0.022 | 0.0257 |

^1^ABX- = samples collected from dogs prior to antibiotic administration; ABX+ = samples collected after receiving metronidazole (20 mg/kg body weight twice daily) for two wk.
^2^SEM = pooled standard errors of the means.
^ab^Mean values within a row with unlike superscript letters differ using parametric analysis (P<0.05).
^yz^Mean values within a row with unlike superscript letters differ using non-parametric analysis (P<0.05).

# **Supplementary Table 3.** Change from baseline (0 hr) pH and SCFA concentrations (μmole/g, OMB) of tubes containing cellulose, pectin, beet pulp, or chicory pulp

|  |  | Δ0 to 6 hr | | Δ0 to 12 hr | | Δ0 to 18 hr | |  | p-value | | |
| --- | --- | --- | --- | --- | --- | --- | --- | --- | --- | --- | --- |
| Fiber |  | ABX-^1^ | ABX+ | ABX- | ABX+ | ABX- | ABX+ | SEM^2^ | Ant | Time | Ant*Time |
| Pectin | pH | -0.48^a^ | -0.79^bc^ | -0.94^d^ | -0.69^b^ | -1.60^e^ | -0.83^cd^ | 0.028 | <0.0001 | <0.0001 | <0.0001 |
|  | Acetate | 754.84^c^ | 369.22^d^ | 1696.50^b^ | 457.18^d^ | 2612.79^a^ | 651.61^c^ | 31.674 | <0.0001 | <0.0001 | <0.0001 |
|  | Propionate | 159.81^c^ | -26.54^d^ | 404.47^b^ | 23.42^d^ | 805.07^a^ | 171.46^c^ | 17.395 | <0.0001 | <0.0001 | <0.0001 |
|  | Butyrate | 69.08^c^ | 23.31^c^ | 415.05^b^ | -13.88^c^ | 1066.09^a^ | -3.53^c^ | 19.040 | <0.0001 | <0.0001 | <0.0001 |
| Beet  pulp | pH | -0.13^a^ | -0.30^b^ | -0.51^d^ | -0.40^c^ | -0.69^e^ | -0.71^e^ | 0.023 | 0.1309 | <0.0001 | <0.0001 |
|  | Acetate | 350.13^d^ | 169.79^d^ | 1256.19^b^ | 742.46^c^ | 1464.61^b^ | 1798.20^a^ | 50.503 | 0.0139 | <0.0001 | <0.0001 |
|  | Propionate | 99.53^c^ | -6.01^d^ | 433.60^b^ | 67.15^cd^ | 560.19^a^ | 447.29^b^ | 20.779 | <0.0001 | <0.0001 | <0.0001 |
|  | Butyrate | 46.47^b^ | 29.99^b^ | 330.89^a^ | 55.69^b^ | 431.61^a^ | 28.14^b^ | 21.317 | <0.0001 | <0.0001 | <0.0001 |
| Chicory pulp | pH | -0.09 | -0.16 | -0.10 | -0.24 | -0.24 | -0.33 | 0.026 | 0.0005 | 0.0001 | 0.4238 |
|  | Acetate | 151.01^b^ | 75.60^b^ | 214.63^b^ | 236.57^b^ | 208.41^b^ | 701.10^a^ | 80.334 | 0.0441 | 0.0041 | 0.0098 |
|  | Propionate | 53.48^bc^ | 3.93^c^ | 76.70^bc^ | -4.81^c^ | 92.45^b^ | 186.48^a^ | 18.769 | 0.4118 | 0.0001 | 0.0013 |
|  | Butyrate | 113.85^ab^ | 11.92^b^ | 124.67^ab^ | 30.36^b^ | 159.82^a^ | -140.46^c^ | 24.575 | <0.0001 | 0.0409 | 0.0017 |
| Cellulose | pH | 0.03^a^ | -0.06^b^ | 0.01^ab^ | 0.03^a^ | 0.00^ab^ | 0.00^ab^ | 0.015 | 0.0836 | 0.1242 | 0.0071 |
|  | Acetate | 24.31 | 9.53 | -35.99 | -0.60 | -19.00 | 40.27 | 34.069 | 0.3570 | 0.5605 | 0.5566 |
|  | Propionate | 7.23^b^ | -2.73^b^ | 1.00^b^ | 35.00^a^ | 5.86^b^ | 5.57^b^ | 8.007 | 0.2492 | 0.1608 | 0.0424 |
|  | Butyrate | 24.21 | 0.71 | -23.45 | 13.92 | -2.46 | -33.28 | 14.018 | 0.6306 | 0.1372 | 0.0609 |

^1^ABX- = samples collected from dogs prior to antibiotic administration; ABX+ = samples collected after receiving metronidazole (20 mg/kg body weight twice daily) for two wk.
^2^SEM = pooled standard errors of the means.
^a-e^Mean values within a row with unlike superscript letters differ using parametric analysis (P<0.05).

# **Supplementary Table 4.** Alpha diversity measures of tubes containing cellulose, pectin, beet pulp, or chicory pulp

|  |  | 0 hr | | 6 hr | | 12 hr | | 18 hr | |  | p-value | | |
| --- | --- | --- | --- | --- | --- | --- | --- | --- | --- | --- | --- | --- | --- |
| Fiber |  | ABX-^1^ | ABX+ | ABX- | ABX+ | ABX- | ABX+ | ABX- | ABX+ | SEM^2^ | Ant | Time | Ant*Time |
| Pectin | Shannon Diversity | 4.76^b^ | 3.83^d^ | 4.95^ab^ | 3.11^f^ | 5.10^a^ | 3.47^e^ | 5.01^ab^ | 4.36^c^ | 0.061 | <0.0001 | <0.0001 | <0.0001 |
|  | Faith's PD | 6.69 | 4.71 | 6.71 | 3.80 | 7.08 | 4.26 | 7.00 | 5.48 | 0.272 | <0.0001 | 0.0189 | 0.0584 |
|  | Pielou Evenness | 0.73^bc^ | 0.71^c^ | 0.76^ab^ | 0.63^d^ | 0.77^a^ | 0.64^d^ | 0.76^ab^ | 0.71^c^ | 0.007 | <0.0001 | <0.0001 | <0.0001 |
| Beet pulp | Shannon Diversity | 4.81^d^ | 3.72^f^ | 5.08^bc^ | 3.67^f^ | 5.23^ab^ | 4.20^e^ | 5.30^a^ | 4.93^cd^ | 0.046 | <0.0001 | <0.0001 | <0.0001 |
|  | Faith's PD | 6.44^a^ | 3.80^d^ | 6.82^a^ | 3.88^d^ | 6.94^a^ | 4.61^c^ | 7.01^a^ | 5.67^b^ | 0.222 | <0.0001 | 0.0003 | 0.0136 |
|  | Pielou Evenness | 0.74^bc^ | 0.72^c^ | 0.77^ab^ | 0.69^d^ | 0.79^a^ | 0.75^bc^ | 0.79^a^ | 0.79^a^ | 0.006 | <0.0001 | <0.0001 | <0.0001 |
| Chicory pulp | Shannon Diversity | 4.83^c^ | 3.69^e^ | 5.14^ab^ | 3.49^e^ | 5.37^a^ | 4.43^d^ | 5.37^a^ | 5.11^b^ | 0.049 | <0.0001 | <0.0001 | <0.0001 |
|  | Faith's PD | 6.79^ab^ | 3.49^d^ | 6.90^ab^ | 3.92^d^ | 7.37^a^ | 4.85^c^ | 7.25^a^ | 6.65^b^ | 0.143 | <0.0001 | <0.0001 | <0.0001 |
|  | Pielou Evenness | 0.74^d^ | 0.71^e^ | 0.77^bc^ | 0.65^f^ | 0.79^abc^ | 0.76^cd^ | 0.79^a^ | 0.77^abc^ | 0.004 | <0.0001 | <0.0001 | <0.0001 |
| Cellulose | Shannon Diversity | 4.77^a^ | 3.60^c^ | 4.78^a^ | 3.67^c^ | 4.83^a^ | 4.32^b^ | 4.89^a^ | 4.79^a^ | 0.087 | <0.0001 | <0.0001 | <0.0001 |
|  | Faith's PD | 6.94^a^ | 2.88^c^ | 6.88^a^ | 4.11^b^ | 7.19^a^ | 4.97^b^ | 7.30^a^ | 6.79^a^ | 0.234 | <0.0001 | <0.0001 | <0.0001 |
|  | Pielou Evenness | 0.74^b^ | 0.74^b^ | 0.74^b^ | 0.69^c^ | 0.74^b^ | 0.78^a^ | 0.75^ab^ | 0.76^ab^ | 0.008 | 0.8235 | <0.0001 | <0.0001 |

^1^ABX- = samples collected from dogs prior to antibiotic administration; ABX+ = samples collected after receiving metronidazole (20 mg/kg body weight twice daily) for two wk.
^2^SEM = pooled standard errors of the means.
^a-f^Mean values within a row with unlike superscript letters differ using parametric analysis (P<0.05).

# **Supplementary Table 5.** Change from baseline (0 hr) bacterial phyla and genera relative abundances (% of sequences) of tubes containing pectin

|  |  | Δ0 to 6 hr | | Δ0 to 12 hr | | Δ0 to 18 hr | |  | p-value | | |
| --- | --- | --- | --- | --- | --- | --- | --- | --- | --- | --- | --- |
| Phyla | Genera | ABX-^1^ | ABX+ | ABX- | ABX+ | ABX- | ABX+ | SEM^2^ | Ant | Time | Ant*Time |
| Actinobacteridota | | 6.70^ab^ | 5.01^abc^ | 4.10^bc^ | 10.32^a^ | 1.87^c^ | 7.00^a^ | 0.892 | 0.0003 | 0.0125 | 0.0006 |
|  | *Adlercreutzia* | -0.02^b^ | 0.00^a^ | -0.11^d^ | 0.00^a^ | -0.05^c^ | 0.00^a^ | 0.004 | <0.0001 | <0.0001 | <0.0001 |
|  | *Bifidobacterium* | 3.68^b^ | 4.02^b^ | 1.69^b^ | 4.77^b^ | 4.71^b^ | 12.89^a^ | 0.822 | <0.0001 | <0.0001 | 0.0009 |
|  | *Collinsella* | 0.53^bc^ | -0.68^d^ | 0.47^bc^ | 0.38^c^ | 0.58^b^ | 0.97^a^ | 0.076 | 0.0003 | <0.0001 | <0.0001 |
|  | *Coriobacteriaceae_UCG-002* | 0.59 | 0.00 | 0.43 | 0.00 | 0.45 | -0.01 | 0.080 | <0.0001 | 0.4433 | 0.4626 |
|  | *Slackia* | -0.11 | 0.00 | -0.08 | 0.00 | -0.05 | 0.00 | 0.005 | 0.0001 | 0.6140 | NA^3^ |
| Bacteroidota | | 6.54^c^ | -2.09^d^ | 14.59^b^ | -2.16^d^ | 21.73^a^ | 1.17^d^ | 0.830 | <0.0001 | <0.0001 | <0.0001 |
|  | *Alloprevotella* | -0.35^c^ | -0.09^a^ | -0.33^c^ | -0.09^a^ | -0.18^b^ | -0.52^d^ | 0.017 | 0.0020 | <0.0001 | <0.0001 |
|  | *Bacteroides* | 5.21^a^ | -0.12^c^ | 4.91^a^ | 2.76^b^ | 3.58^b^ | -5.46^d^ | 0.376 | <0.0001 | <0.0001 | <0.0001 |
|  | *Muribaculaceae* | 1.74 | -0.02 | 0.72 | -0.03 | 0.63 | -0.49 | 0.194 | <0.0001 | 0.0042 | 0.0549 |
|  | *Parabacteroides* | 0.04^a^ | 0.00^ab^ | -0.05^bc^ | 0.01^ab^ | -0.19^d^ | -0.08^c^ | 0.016 | 0.0064 | <0.0001 | 0.0027 |
|  | *Prevotella* | 8.72 | -0.01 | 18.00 | 1.28 | 0.46 | -0.13 | 0.469 | 0.0053 | 0.0138 | NA |
|  | *Prevotellaceae_Ga6A1_group* | 0.00^b^ | 0.00^b^ | -0.01^b^ | 0.00^b^ | 0.09^a^ | 0.00^b^ | 0.007 | 0.0002 | <0.0001 | <0.0001 |
|  | *Rikenellaceae_RC9_gut_group* | -0.07^bc^ | 0.00^a^ | -0.03^ab^ | 0.00^a^ | -0.06^bc^ | -0.08^c^ | 0.010 | 0.0107 | 0.0003 | 0.0019 |
| Firmicutes | | 5.24^c^ | 28.06^b^ | 1.66^c^ | 36.87^a^ | -6.90^d^ | 25.59^b^ | 1.202 | <0.0001 | <0.0001 | 0.0006 |
|  | *[Eubacterium]_brachy_group* | -1.97^c^ | 0.02^b^ | -2.23^d^ | 0.38^a^ | -2.52^e^ | -2.21^d^ | 0.051 | <0.0001 | <0.0001 | <0.0001 |
|  | *[Eubacterium]_nodatum_group* | -0.16 | 0.00 | -0.08 | 0.00 | -0.27 | -0.63 | 0.023 | 0.2313 | 0.0028 | NA |
|  | *[Ruminococcus]_gauvreauii_group* | 0.22 | 0.00 | 0.17 | 0.00 | 0.09 | 0.00 | 0.028 | <0.0001 | 0.0579 | 0.0579 |
|  | *[Ruminococcus]_gnavus_group* | 0.23 | 0.03 | 0.21 | 0.02 | 0.59 | 0.04 | 0.054 | 0.0003 | 0.3160 | NA |
|  | *[Ruminococcus]_torques_group* | 0.50^b^ | -0.06^d^ | 0.19^c^ | -0.15^e^ | 0.70^a^ | -0.21^e^ | 0.019 | <0.0001 | <0.0001 | <0.0001 |
|  | *Allobaculum* | -3.03^c^ | -2.22^c^ | 0.32^b^ | 0.89^b^ | -5.49^d^ | 3.86^a^ | 0.463 | <0.0001 | <0.0001 | <0.0001 |
|  | *Anaeroplasma* | -0.01^a^ | -0.44^b^ | 0.04^a^ | -0.79^c^ | -0.01^a^ | -0.73^c^ | 0.039 | <0.0001 | 0.0020 | 0.0004 |
|  | *Blautia* | 2.17^b^ | 0.04^d^ | 1.52^c^ | 0.19^d^ | 3.15^a^ | -0.72^e^ | 0.112 | <0.0001 | 0.0234 | <0.0001 |
|  | *Butyricicoccus* | 0.06 | 0.02 | 0.02 | -0.02 | 0.09 | -0.06 | 0.028 | 0.0069 | 0.2033 | NA |
|  | *Clostridium_sensu_stricto_1* | -3.25^c^ | 0.03^a^ | -3.45^c^ | -0.86^a^ | -3.40^c^ | -2.13^b^ | 0.160 | <0.0001 | 0.0010 | 0.0116 |
|  | *Dubosiella* | 1.34^ab^ | -0.70^c^ | 0.45^bc^ | -0.38^c^ | 2.42^a^ | -0.33^c^ | 0.283 | <0.0001 | 0.0129 | 0.0187 |
|  | *Enterococcus* | 0.63^c^ | 9.65^b^ | 0.52^c^ | 14.58^b^ | 0.50^c^ | 30.26^a^ | 1.399 | <0.0001 | <0.0001 | <0.0001 |
|  | Erysipelatoclostridiaceae uncultured | -2.06^b^ | -0.22^a^ | -5.58^c^ | 0.04^a^ | 0.13^a^ | 0.05^a^ | 0.319 | <0.0001 | <0.0001 | <0.0001 |
|  | *Faecalibacterium* | 3.97^b^ | -0.04^d^ | 6.05^a^ | 1.08^c^ | 0.72^c^ | -0.09^d^ | 0.211 | <0.0001 | <0.0001 | <0.0001 |
|  | *Faecalibaculum* | 0.29^ab^ | -0.03^c^ | 0.06^bc^ | -0.03^c^ | 0.40^a^ | -0.03^c^ | 0.055 | <0.0001 | 0.0139 | 0.0139 |
|  | *Lachnoclostridium* | 0.05^bc^ | 0.00^cd^ | 0.18^a^ | 0.11^ab^ | -0.22^e^ | -0.08^d^ | 0.024 | 0.7894 | <0.0001 | 0.0012 |
|  | Lachnospiraceae unclassified | 0.25^ab^ | 0.00^bc^ | -0.28^c^ | 0.42^a^ | 0.16^ab^ | -0.65^c^ | 0.059 | 0.0270 | 0.0001 | <0.0001 |
|  | Lachnospiraceae uncultured | -0.07^bc^ | 0.00^b^ | 0.07^b^ | 0.28^a^ | -0.14^c^ | -0.34^d^ | 0.034 | 0.3646 | <0.0001 | 0.0002 |
|  | *Lactobacillus* | 0.06^c^ | 2.90^b^ | -0.03^c^ | 3.57^ab^ | 0.00^c^ | 4.36^a^ | 0.171 | <0.0001 | 0.0048 | 0.0030 |
|  | *Megamonas* | -0.01 | 0.00 | -0.05 | 0.00 | -0.25 | 0.00 | 0.013 | 0.0338 | 0.1341 | NA |
|  | *Negativibacillus* | -0.36 | 0.00 | -0.56 | 0.00 | -0.57 | -0.49 | 0.012 | 0.0045 | 0.0426 | NA |
|  | *Oribacterium* | -0.60^b^ | 0.00^a^ | -0.60^b^ | 0.09^a^ | -0.81^c^ | -1.06^d^ | 0.023 | <0.0001 | <0.0001 | <0.0001 |
|  | *Peptoclostridium* | 6.47^ab^ | 0.70^c^ | 4.38^b^ | -3.96^d^ | 8.61^a^ | -4.23^e^ | 0.442 | <0.0001 | <0.0001 | <0.0001 |
|  | *Peptococcus* | -0.08^c^ | 0.00^a^ | -0.15^e^ | -0.03^b^ | -0.12^d^ | -0.39^f^ | 0.005 | 0.0002 | <0.0001 | <0.0001 |
|  | *Peptostreptococcus* | -4.42^b^ | -0.12^a^ | -6.40^c^ | -4.96^b^ | -5.20^b^ | -6.57^c^ | 0.307 | <0.0001 | <0.0001 | <0.0001 |
|  | *Phascolarctobacterium* | 0.21^b^ | 0.21^b^ | 0.50^a^ | -0.43^c^ | 0.09^b^ | -0.76^d^ | 0.045 | <0.0001 | <0.0001 | <0.0001 |
|  | *Romboutsia* | -0.44 | 0.00 | -0.41 | 0.00 | -0.40 | 0.00 | 0.013 | <0.0001 | 0.3605 | 0.3605 |
|  | *Sellimonas* | -0.09 | 0.00 | -0.16 | -0.03 | -0.14 | -0.37 | 0.016 | 0.2303 | 0.0150 | NA |
|  | *Streptococcus* | 2.30 | 19.52 | 2.04 | 16.75 | 3.66 | 20.85 | 0.685 | <0.0001 | 0.0055 | 0.1490 |
|  | *Terrisporobacter* | 0.00 | -1.33 | 0.00 | -0.35 | 0.00 | -0.06 | 0.012 | 0.0001 | 0.5824 | NA |
|  | *Turicibacter* | -0.04 | -0.09 | -0.03 | -0.09 | -0.02 | -0.09 | 0.006 | 0.0001 | 0.9413 | NA |
|  | *UCG-005* | -0.20 | 0.00 | -0.17 | 0.00 | -0.23 | -0.14 | 0.008 | 0.0003 | 0.2527 | NA |
| Fusobacteriota | | -16.22^c^ | -0.18^a^ | -16.97^c^ | -24.33^e^ | -13.34^b^ | -20.47^d^ | 0.420 | 0.1577 | <0.0001 | <0.0001 |
|  | *Cetobacterium* | 0.12 | -0.02 | 0.09 | -0.02 | 0.51 | -0.02 | 0.011 | 0.0001 | 0.6394 | NA |
|  | *Fusobacterium* | -19.69^c^ | 1.00^a^ | -18.98^c^ | -17.90^c^ | -8.58^b^ | -28.01^d^ | 0.411 | 0.0389 | <0.0001 | <0.0001 |
| Proteobacteria | | -2.25^a^ | -30.79^d^ | -3.36^a^ | -20.68^c^ | -3.37^a^ | -13.27^b^ | 0.570 | <0.0001 | <0.0001 | <0.0001 |
|  | *Anaerobiospirillum* | -0.06^bc^ | -0.13^bc^ | -0.01^b^ | 0.16^a^ | 0.01^b^ | -0.20^c^ | 0.030 | 0.1762 | 0.0001 | 0.0002 |
|  | *Escherichia-Shigella* | 0.02 | -4.59 | 0.00 | -5.99 | -0.02 | -22.66 | 0.510 | 0.0003 | 0.9599 | NA |
|  | *Morganella* | 0.00 | -1.24 | 0.00 | -2.38 | 0.00 | -1.01 | 0.005 | 0.0001 | 0.5805 | NA |
|  | *Parasutterella* | -0.95^b^ | -1.35^c^ | -0.80^ab^ | -0.83^b^ | -0.64^a^ | -0.95^b^ | 0.042 | <0.0001 | <0.0001 | 0.0006 |
|  | *Proteus* | 0.00^a^ | -2.35^c^ | 0.00^a^ | -1.66^b^ | -1.57^b^ | 0.00^a^ | 0.028 | <0.0001 | <0.0001 | <0.0001 |

^1^ABX- = samples collected from dogs prior to antibiotic administration; ABX+ = samples collected after receiving metronidazole (20 mg/kg body weight twice daily) for two wk.
^2^SEM = pooled standard errors of the means.
^3^NA: data not normal, requiring non-parametric statistical analysis.
^a-f^Mean values within a row with unlike superscript letters differ using parametric analysis (P<0.05).

# **Supplementary Table 6.** Change from baseline (0 hr) bacterial phyla and genera relative abundances (% of sequences) of tubes containing beet pulp

|  |  | Δ0 to 6 hr | | Δ0 to 12 hr | | Δ0 to 18 hr | |  | p-value | | |
| --- | --- | --- | --- | --- | --- | --- | --- | --- | --- | --- | --- |
| Phyla | Genera | ABX-^1^ | ABX+ | ABX- | ABX+ | ABX- | ABX+ | SEM^2^ | Ant | Time | Ant*Time |
| Actinobacteridota | | 9.71^a^ | 4.73^abc^ | 1.52^bc^ | 8.94^a^ | 1.24^c^ | 7.52^ab^ | 2.667 | 0.0011 | 0.2198 | 0.0069 |
|  | *Adlercreutzia* | -0.02 | 0.00 | -0.09 | 0.00 | -0.03 | 0.00 | 0.011 | 0.0029 | 0.4367 | NA^3^ |
|  | *Bifidobacterium* | 1.77^c^ | 3.21^c^ | 1.52^c^ | 5.93^b^ | 1.19^c^ | 12.59^a^ | 0.519 | <0.0001 | <0.0001 | <0.0001 |
|  | *Collinsella* | 0.28^bc^ | -1.26^d^ | 0.32^b^ | -0.27^c^ | 0.50^ab^ | 0.97^a^ | 0.171 | 0.0002 | <0.0001 | <0.0001 |
|  | *Coriobacteriaceae_UCG-002* | 0.07 | 0.00 | 0.03 | 0.00 | 0.07 | -0.01 | 0.035 | 0.0411 | 0.7441 | 0.6644 |
|  | *Slackia* | -0.03 | 0.00 | -0.07 | 0.01 | 0.00 | 0.00 | 0.007 | 0.0002 | 0.7379 | NA |
| Bacteroidota | | 3.40 | -1.52 | 14.83 | 18.26 | 13.45 | 18.62 | 2.054 | 0.1451 | 0.0032 | NA |
|  | *Alloprevotella* | -0.27^c^ | -0.06^b^ | -0.26^c^ | -0.04^b^ | 0.05^a^ | -0.45^d^ | 0.017 | 0.1494 | 0.0393 | <0.0001 |
|  | *Bacteroides* | 9.97^c^ | 20.01^a^ | 10.72^bc^ | 13.63^b^ | 5.78^d^ | -5.02^e^ | 0.667 | 0.2128 | <0.0001 | <0.0001 |
|  | *Muribaculaceae* | 0.04 | 0.01 | -0.44 | 0.05 | -1.25 | -0.47 | 0.207 | 0.0104 | 0.0007 | 0.0774 |
|  | *Parabacteroides* | 0.06 | 0.00 | 0.01 | 0.02 | -0.11 | -0.08 | 0.029 | 0.8939 | 0.0082 | NA |
|  | *Prevotella* | 5.63^b^ | 0.23^d^ | 4.77^b^ | 7.70^a^ | 0.56^c^ | -0.10^e^ | 0.347 | <0.0001 | <0.0001 | <0.0001 |
|  | *Prevotellaceae_Ga6A1_group* | -0.02 | 0.00 | -0.03 | 0.00 | 0.10 | 0.00 | 0.008 | 0.6710 | 0.0472 | NA |
|  | *Rikenellaceae_RC9_gut_group* | -0.06 | 0.00 | -0.07 | 0.00 | -0.04 | -0.08 | 0.015 | 0.3439 | 0.2271 | NA |
| Firmicutes | | 4.76^b^ | 15.10^a^ | 2.96^b^ | 4.40^b^ | 0.59^b^ | 6.40^b^ | 1.324 | 0.0002 | 0.0006 | 0.0177 |
|  | *[Eubacterium]_brachy_group* | -1.72^c^ | 0.11^b^ | -1.78^c^ | 0.50^a^ | -1.88^cd^ | -2.21^d^ | 0.082 | <0.0001 | <0.0001 | <0.0001 |
|  | *[Eubacterium]_nodatum_group* | -0.22^c^ | 0.00^b^ | -0.16^c^ | 0.19^a^ | -0.31^d^ | -0.63^e^ | 0.020 | 0.0002 | <0.0001 | <0.0001 |
|  | *[Ruminococcus]_gauvreauii_group* | 0.14 | 0.00 | 0.20 | 0.00 | 0.05 | 0.00 | 0.010 | 0.0001 | 0.5824 | NA |
|  | *[Ruminococcus]_gnavus_group* | -0.31^d^ | 0.03^b^ | -0.11^c^ | 0.05^ab^ | 0.03^ab^ | 0.08^a^ | 0.013 | <0.0001 | <0.0001 | <0.0001 |
|  | *[Ruminococcus]_torques_group* | 0.29 | -0.06 | 0.43 | -0.01 | 0.23 | -0.20 | 0.037 | <0.0001 | 0.0006 | 0.3989 |
|  | *Allobaculum* | -4.57^cd^ | -5.64^d^ | -3.91^c^ | -2.26^b^ | -5.02^cd^ | 4.76^a^ | 0.277 | <0.0001 | <0.0001 | <0.0001 |
|  | *Anaeroplasma* | 0.01^a^ | -0.28^b^ | 0.00^a^ | -0.67^c^ | 0.05^a^ | 0.02^a^ | 0.039 | <0.0001 | <0.0001 | <0.0001 |
|  | *Blautia* | 2.73^a^ | 0.10^d^ | 2.90^a^ | 0.94^c^ | 2.15^b^ | -0.65^e^ | 0.121 | <0.0001 | <0.0001 | 0.0049 |
|  | *Butyricicoccus* | 0.03^a^ | 0.03^a^ | 0.02^a^ | 0.03^a^ | 0.03^a^ | -0.06^b^ | 0.013 | 0.0307 | 0.0034 | 0.0032 |
|  | *Clostridium_sensu_stricto_1* | -2.68 | 3.45 | -2.67 | 0.71 | -2.73 | -2.00 | 0.556 | 0.0003 | 0.5195 | NA |
|  | *Dubosiella* | 0.89 | -0.31 | 0.82 | -0.16 | 1.03 | 0.06 | 0.128 | <0.0001 | 0.0200 | 0.0703 |
|  | *Enterococcus* | 0.23 | -10.41 | 0.42 | 0.71 | 0.24 | 22.15 | 1.421 | 0.6911 | 0.0292 | NA |
|  | Erysipelotrichaceae uncultured | -1.53^c^ | -0.24^ab^ | -1.55^c^ | -0.05^a^ | -0.66^b^ | 0.11^a^ | 0.138 | <0.0001 | 0.0007 | 0.0286 |
|  | *Faecalibacterium* | 4.25^b^ | 0.04^d^ | 4.63^b^ | 6.09^a^ | 1.28^c^ | -0.06^d^ | 0.212 | <0.0001 | <0.0001 | <0.0001 |
|  | *Faecalibaculum* | 0.08 | 0.00 | 0.06 | 0.03 | 0.00 | 0.00 | 0.053 | 0.3774 | 0.6096 | 0.7477 |
|  | *Lachnoclostridium* | 0.10^b^ | 0.00^bc^ | 0.14^ab^ | 0.28^a^ | -0.13^c^ | -0.08^c^ | 0.036 | 0.3125 | <0.0001 | 0.0202 |
|  | Lachnospiraceae unclassified | 2.34^a^ | 0.02^c^ | 1.99^a^ | 1.04^b^ | 0.58^bc^ | -0.65^d^ | 0.122 | <0.0001 | <0.0001 | 0.0003 |
|  | Lachnospiraceae uncultured | 0.00^c^ | 0.00^c^ | 0.25^b^ | 0.56^a^ | 0.00^c^ | -0.34^d^ | 0.049 | 0.7359 | <0.0001 | <0.0001 |
|  | *Lactobacillus* | 0.08^c^ | 0.50^c^ | 0.01^c^ | 1.65^b^ | 0.06^c^ | 4.54^a^ | 0.280 | <0.0001 | 0.0001 | <0.0001 |
|  | *Megamonas* | 0.02 | 0.00 | -0.06 | 0.00 | -0.21 | 0.00 | 0.010 | 0.0337 | 0.0958 | NA |
|  | *Negativibacillus* | -0.27 | 0.00 | -0.46 | 0.00 | -0.47 | -0.49 | 0.010 | 0.2237 | 0.0055 | NA |
|  | *Oribacterium* | -0.46^c^ | 0.05^b^ | -0.40^c^ | 0.29^a^ | -0.61^d^ | -1.06^e^ | 0.031 | <0.0001 | <0.0001 | <0.0001 |
|  | *Peptoclostridium* | 4.38^b^ | 1.08^c^ | 4.90^b^ | -3.67^d^ | 7.08^a^ | -4.06^d^ | 0.441 | <0.0001 | 0.0016 | <0.0001 |
|  | *Peptococcus* | -0.04^bc^ | 0.00^b^ | -0.07^c^ | 0.08^a^ | -0.02^bc^ | -0.39^d^ | 0.011 | <0.0001 | <0.0001 | <0.0001 |
|  | *Peptostreptococcus* | -3.20^b^ | 2.47^a^ | -4.46^bc^ | -4.19^b^ | -3.33^b^ | -6.49^c^ | 0.479 | 0.0354 | <0.0001 | <0.0001 |
|  | *Phascolarctobacterium* | 0.48^a^ | 0.26^ab^ | 0.42^a^ | 0.16^bc^ | -0.02^c^ | -0.76^d^ | 0.052 | <0.0001 | <0.0001 | <0.0001 |
|  | *Romboutsia* | -0.13 | 0.00 | -0.12 | 0.00 | -0.11 | 0.00 | 0.021 | <0.0001 | 0.9081 | 0.9081 |
|  | *Sellimonas* | -0.04^ab^ | 0.00^a^ | -0.11^b^ | 0.01^a^ | -0.12^b^ | -0.37^c^ | 0.017 | 0.0705 | <0.0001 | <0.0001 |
|  | *Streptococcus* | 2.31^d^ | 4.98^b^ | 2.12^d^ | 4.45^bc^ | 2.89^cd^ | 12.22^a^ | 0.371 | <0.0001 | <0.0001 | <0.0001 |
|  | *Terrisporobacter* | 0.00^c^ | -0.87^d^ | 0.00^c^ | 0.18^b^ | 0.00^c^ | 0.68^a^ | 0.014 | 0.7325 | <0.0001 | <0.0001 |
|  | *Turicibacter* | -0.02 | 0.03 | 0.00 | 0.03 | 0.01 | 0.03 | 0.010 | 0.0009 | 0.2764 | 0.2764 |
|  | *UCG-005* | -0.18 | 0.00 | -0.14 | 0.00 | -0.21 | -0.14 | 0.007 | 0.0007 | 0.1070 | NA |
| Fusobacteriota | | -19.66 | -0.05 | -15.34 | -16.35 | -11.49 | -17.81 | 2.990 | 0.6272 | 0.1576 | NA |
|  | *Cetobacterium* | 0.10^b^ | 0.00^c^ | 0.15^b^ | 0.00^c^ | 0.53^a^ | 0.00^c^ | 0.014 | <0.0001 | <0.0001 | <0.0001 |
|  | *Fusobacterium* | -18.03^c^ | 8.95^a^ | -17.18^c^ | -15.25^c^ | -4.26^b^ | -27.90^d^ | 0.606 | 0.0040 | <0.0001 | <0.0001 |
| Proteobacteria | | 1.78 | -18.24 | -3.95 | -15.23 | -3.81 | -14.72 | 1.606 | 0.0003 | 0.9487 | NA |
|  | *Anaerobiospirillum* | 0.01 | 0.26 | 0.03 | -0.01 | 0.05 | -0.07 | 0.058 | 0.4529 | 0.2714 | NA |
|  | *Escherichia-Shigella* | -0.03 | -23.81 | -0.04 | -9.87 | 0.03 | 2.74 | 0.194 | 0.2332 | 0.0031 | NA |
|  | *Morganella* | 0.00 | -0.74 | 0.00 | -2.22 | 0.00 | -0.47 | 0.053 | 0.0001 | 0.6394 | NA |
|  | *Parasutterella* | -1.40^d^ | -0.79^c^ | -1.35^d^ | -0.47^ab^ | -0.67^bc^ | -0.28^a^ | 0.052 | <0.0001 | <0.0001 | 0.0024 |
|  | *Proteus* | 0.00 | -1.62 | 0.00 | -1.50 | 0.00 | -0.16 | 0.048 | 0.0030 | 0.2832 | NA |

^1^ABX- = samples collected from dogs prior to antibiotic administration; ABX+ = samples collected after receiving metronidazole (20 mg/kg body weight twice daily) for two wk.
^2^SEM = pooled standard errors of the means.
^3^NA: data not normal, requiring non-parametric statistical analysis.
^a-e^Mean values within a row with unlike superscript letters differ using parametric analysis (P<0.05).

# **Supplementary Table 7.** Change from baseline (0 hr) bacterial phyla and genera relative abundances (% of sequences) of tubes containing chicory pulp

|  |  | Δ0 to 6 hr | | Δ0 to 12 hr | | Δ0 to 18 hr | |  | p-value | | |
| --- | --- | --- | --- | --- | --- | --- | --- | --- | --- | --- | --- |
| Phyla | Genera | ABX-^1^ | ABX+ | ABX- | ABX+ | ABX- | ABX+ | SEM^2^ | Ant | Time | Ant*Time |
| Actinobacteridota | | 0.04^a^ | -7.17^d^ | -0.37^a^ | -4.39^c^ | -0.68^a^ | -2.77^b^ | 0.438 | <0.0001 | 0.0005 | <0.0001 |
|  | *Adlercreutzia* | 0.01 | 0.00 | -0.02 | 0.00 | -0.01 | 0.00 | 0.013 | 0.5429 | 0.5982 | 0.5982 |
|  | *Bifidobacterium* | -0.11^ab^ | -9.60^d^ | -0.15^ab^ | -4.23^c^ | -0.61^b^ | 1.06^a^ | 0.389 | <0.0001 | <0.0001 | <0.0001 |
|  | *Collinsella* | 0.23^b^ | -1.76^d^ | 0.11^b^ | -0.43^c^ | -0.21^c^ | 0.62^a^ | 0.072 | <0.0001 | <0.0001 | <0.0001 |
|  | *Coriobacteriaceae_UCG-002* | 0.04^a^ | 0.00^a^ | -0.09^ab^ | 0.00^a^ | -0.19^b^ | -0.01^ab^ | 0.041 | 0.0333 | 0.0332 | 0.0417 |
|  | *Slackia* | -0.01^b^ | 0.01^ab^ | -0.01^b^ | 0.05^a^ | -0.02^b^ | 0.02^ab^ | 0.012 | 0.0014 | 0.1118 | 0.1414 |
| Bacteroidota | | 4.68^c^ | -1.18^d^ | 7.93^ab^ | 6.79^bc^ | 5.88^bc^ | 10.08^a^ | 0.647 | 0.0963 | <0.0001 | <0.0001 |
|  | *Alloprevotella* | -0.34^c^ | -0.01^b^ | -0.31^c^ | 0.13^a^ | -0.08^b^ | -0.44^c^ | 0.029 | 0.0001 | 0.0004 | <0.0001 |
|  | *Bacteroides* | 7.31^a^ | 7.99^a^ | 7.00^a^ | 8.68^a^ | 3.60^b^ | -4.70^c^ | 0.434 | 0.0001 | <0.0001 | <0.0001 |
|  | *Muribaculaceae* | 0.53 | 0.05 | -0.44 | 0.41 | -1.67 | -0.47 | 0.231 | 0.3528 | 0.0059 | NA^3^ |
|  | *Parabacteroides* | 0.06^b^ | 0.00^c^ | 0.05^b^ | 0.10^a^ | -0.19^e^ | -0.08^d^ | 0.008 | 0.0004 | <0.0001 | <0.0001 |
|  | *Prevotella* | 1.00^ab^ | 0.68^b^ | 0.97^b^ | 3.47^a^ | 0.64^b^ | -0.08^c^ | 0.141 | 0.0012 | <0.0001 | <0.0001 |
|  | *Prevotellaceae_Ga6A1_group* | 0.00^b^ | 0.00^b^ | -0.01^b^ | 0.00^b^ | 0.11^a^ | 0.00^b^ | 0.010 | 0.0012 | 0.0002 | 0.0002 |
|  | *RikenellaceaeRC9_gut_group* | -0.07^bc^ | 0.00^a^ | -0.06^b^ | 0.01^a^ | -0.03^ab^ | -0.08^c^ | 0.009 | 0.0034 | 0.0253 | <0.0001 |
| Firmicutes | | 4.22^c^ | 24.02^a^ | 3.65^c^ | 14.64^b^ | 4.16^c^ | 15.97^b^ | 1.039 | <0.0001 | 0.0007 | 0.0014 |
|  | *[Eubacterium]_brachy_group* | -1.08^c^ | 0.13^b^ | -0.87^c^ | 1.60^a^ | -1.87^d^ | -2.21^d^ | 0.118 | <0.0001 | <0.0001 | <0.0001 |
|  | *[Eubacterium]_nodatum_group* | -0.18^d^ | 0.00^b^ | -0.10^c^ | 0.49^a^ | -0.31^e^ | -0.63^f^ | 0.017 | <0.0001 | <0.0001 | <0.0001 |
|  | *[Ruminococcus]_gauvreauii_group* | 0.18 | 0.00 | 0.13 | 0.00 | 0.20 | 0.00 | 0.013 | 0.0001 | 0.6567 | NA |
|  | *[Ruminococcus]_gnavus_group* | 0.08^b^ | 0.02^b^ | 0.11^b^ | 0.04^b^ | 0.53^a^ | 0.08^b^ | 0.042 | <0.0001 | <0.0001 | 0.0003 |
|  | *[Ruminococcus]_torques_group* | 0.21^ab^ | 0.05^bc^ | 0.20^ab^ | 0.43^a^ | 0.31^a^ | -0.17^c^ | 0.049 | 0.0074 | 0.0015 | <0.0001 |
|  | *Allobaculum* | -2.51^b^ | -5.33^c^ | -2.52^b^ | -1.67^b^ | -3.07^b^ | 3.38^a^ | 0.270 | <0.0001 | <0.0001 | <0.0001 |
|  | *Anaeroplasma* | 0.00^a^ | -0.09^a^ | -0.01^a^ | -0.18^b^ | -0.01^a^ | -0.37^c^ | 0.018 | <0.0001 | <0.0001 | <0.0001 |
|  | *Blautia* | 3.26^a^ | 0.09^d^ | 2.61^b^ | 1.01^c^ | 3.03^ab^ | -0.64^e^ | 0.116 | <0.0001 | 0.0005 | <0.0001 |
|  | *Butyricicoccus* | 0.09 | 0.07 | 0.12 | 0.09 | 0.09 | -0.06 | 0.031 | 0.0375 | 0.2069 | NA |
|  | *Clostridium_sensu_stricto_1* | -1.60^c^ | 4.26^a^ | -1.06^c^ | 0.80^b^ | -1.63^c^ | -1.93^c^ | 0.176 | <0.0001 | <0.0001 | <0.0001 |
|  | *Dubosiella* | 0.79^b^ | -0.41^d^ | 1.20^a^ | -0.05^c^ | 0.97^ab^ | 0.09^c^ | 0.059 | <0.0001 | 0.0001 | 0.0180 |
|  | *Enterococcus* | 1.60^c^ | -1.40^d^ | 2.39^c^ | 9.71^b^ | 1.03^cd^ | 35.38^a^ | 0.558 | <0.0001 | <0.0001 | <0.0001 |
|  | Erysipelotrichaceae uncultured | 0.54^a^ | -0.21^b^ | 0.24^ab^ | 0.03^ab^ | -0.18^b^ | 0.27^ab^ | 0.143 | 0.1497 | 0.6320 | 0.0037 |
|  | *Faecalibacterium* | 0.54^a^ | 0.04^c^ | 0.60^a^ | 0.52^a^ | 0.32^b^ | -0.06^c^ | 0.045 | <0.0001 | <0.0001 | 0.0009 |
|  | *Faecalibaculum* | 0.28^a^ | 0.00^b^ | 0.47^a^ | 0.02^b^ | 0.07^b^ | 0.00^b^ | 0.048 | <0.0001 | 0.0012 | 0.0023 |
|  | *Lachnoclostridium* | 0.01 | 0.00 | 0.12 | 0.15 | -0.15 | -0.08 | 0.020 | 0.1273 | <0.0001 | 0.1567 |
|  | Lachnospiraceae unclassified | 0.33^b^ | 0.00^d^ | 0.19^c^ | 0.57^a^ | 0.29^bc^ | -0.65^e^ | 0.026 | <0.0001 | <0.0001 | <0.0001 |
|  | Lachnospiraceae uncultured | 0.13^b^ | 0.02^b^ | 0.13^ab^ | 0.27^a^ | 0.12^b^ | -0.34^c^ | 0.029 | <0.0001 | <0.0001 | <0.0001 |
|  | *Lactobacillus* | 0.04 | 0.41 | 0.04 | 1.59 | 0.05 | 4.45 | 0.187 | 0.0054 | 0.2148 | NA |
|  | *Megamonas* | 0.03^b^ | 0.00^b^ | 0.07^a^ | 0.00^b^ | -0.22^c^ | 0.00^b^ | 0.010 | 0.0001 | <0.0001 | <0.0001 |
|  | *Negativibacillus* | -0.12^c^ | 0.00^b^ | -0.31^d^ | 0.12^a^ | -0.43^e^ | -0.49^e^ | 0.015 | <0.0001 | <0.0001 | <0.0001 |
|  | *Oribacterium* | -0.36^d^ | 0.06^b^ | -0.21^c^ | 0.49^a^ | -0.61^e^ | -1.06^f^ | 0.032 | <0.0001 | <0.0001 | <0.0001 |
|  | *Peptoclostridium* | 2.87^a^ | 3.23^a^ | 4.97^a^ | -1.20^b^ | 4.08^a^ | -4.01^c^ | 0.548 | <0.0001 | 0.0007 | <0.0001 |
|  | *Peptococcus* | 0.06^b^ | 0.00^c^ | 0.01^c^ | 0.22^a^ | 0.01^c^ | -0.39^d^ | 0.008 | <0.0001 | <0.0001 | <0.0001 |
|  | *Peptostreptococcus* | -2.91^b^ | 2.22^a^ | -3.12^b^ | -2.25^b^ | -3.38^b^ | -6.49^c^ | 0.490 | 0.0002 | <0.0001 | <0.0001 |
|  | *Phascolarctobacterium* | 0.10^ab^ | 0.21^a^ | 0.16^a^ | 0.03^b^ | 0.00^b^ | -0.76^c^ | 0.042 | <0.0001 | <0.0001 | <0.0001 |
|  | *Romboutsia* | -0.19^b^ | 0.00^a^ | -0.13^ab^ | 0.00^a^ | -0.16^b^ | 0.00^a^ | 0.028 | 0.0001 | 0.8692 | NA |
|  | *Sellimonas* | 0.04^b^ | 0.01^b^ | -0.05^b^ | 0.19^a^ | 0.01^b^ | -0.37^c^ | 0.021 | 0.0071 | <0.0001 | <0.0001 |
|  | *Streptococcus* | 1.52^c^ | 3.07^b^ | 1.75^c^ | 3.34^b^ | 1.47^c^ | 9.19^a^ | 0.255 | <0.0001 | <0.0001 | <0.0001 |
|  | *Terrisporobacter* | 0.00 | -0.92 | 0.00 | 0.04 | 0.00 | 0.71 | 0.032 | 0.6712 | 0.0326 | NA |
|  | *Turicibacter* | -0.03 | -0.02 | -0.03 | -0.02 | -0.03 | -0.02 | 0.008 | 0.0333 | 0.8266 | NA |
|  | *UCG-005* | -0.01^cd^ | 0.00^bc^ | 0.23^a^ | 0.11^b^ | -0.12^de^ | -0.14^e^ | 0.028 | 0.0529 | <0.0001 | 0.0479 |
| Fusobacteriota | | -6.87^b^ | -0.07^a^ | -9.08^b^ | -7.86^b^ | -7.21^b^ | -14.54^c^ | 0.578 | 0.6393 | <0.0001 | <0.0001 |
|  | *Cetobacterium* | 0.37 | 0.00 | 0.25 | 0.00 | 0.75 | 0.00 | 0.025 | 0.0001 | 0.6143 | NA |
|  | *Fusobacterium* | -12.05^c^ | 17.44^a^ | -13.01^c^ | -11.99^c^ | 0.53^b^ | -27.93^d^ | 0.577 | 0.1719 | <0.0001 | <0.0001 |
| Proteobacteria | | -2.12^a^ | -15.61^c^ | -2.12^a^ | -9.17^b^ | -2.15^a^ | -8.75^b^ | 0.465 | <0.0001 | <0.0001 | <0.0001 |
|  | *Anaerobiospirillum* | -0.03^bc^ | 0.21^a^ | 0.01^b^ | -0.13^d^ | 0.04^b^ | -0.09^cd^ | 0.017 | 0.6135 | <0.0001 | <0.0001 |
|  | *Escherichia-Shigella* | 0.01 | -21.59 | -0.02 | -7.55 | -0.01 | 4.51 | 0.371 | 0.2332 | 0.0319 | NA |
|  | *Morganella* | 0.00 | -0.05 | 0.00 | -2.06 | 0.00 | -0.29 | 0.143 | 0.0338 | 0.1553 | NA |
|  | *Parasutterella* | -0.97 | -0.46 | -1.03 | -0.23 | -0.75 | -0.12 | 0.091 | <0.0001 | 0.0050 | 0.1319 |
|  | *Proteus* | 0.00 | -0.23 | 0.00 | -1.15 | 0.00 | -1.36 | 0.050 | 0.0001 | 0.5824 | NA |

^1^ABX- = samples collected from dogs prior to antibiotic administration; ABX+ = samples collected after receiving metronidazole (20 mg/kg body weight twice daily) for two wk.
^2^SEM = pooled standard errors of the means.
^3^NA: data not normal, requiring non-parametric statistical analysis.
^a-e^Mean values within a row with unlike superscript letters differ using parametric analysis (P<0.05).

# **Supplementary Table 8.** Change from baseline (0 hr) bacterial phyla and genera relative abundances (% of sequences) of tubes containing cellulose

|  |  | Δ0 to 6 hr | | Δ0 to 12 hr | | Δ0 to 18 hr | |  | p-value | | |
| --- | --- | --- | --- | --- | --- | --- | --- | --- | --- | --- | --- |
| Phyla | Genera | ABX-^1^ | ABX+ | ABX- | ABX+ | ABX- | ABX+ | SEM^2^ | Ant | Time | Ant*Time |
| Actinobacteridota | | 0.18 | -4.24 | -0.73 | -5.39 | -0.75 | -5.28 | 0.622 | <0.0001 | 0.0537 | 0.9567 |
|  | *Adlercreutzia* | 0.02 | 0.00 | -0.01 | 0.00 | -0.01 | 0.00 | 0.014 | 0.8806 | 0.5189 | 0.5189 |
|  | *Bifidobacterium* | -0.24^b^ | -11.23^d^ | -0.13^b^ | -7.07^c^ | -0.76^b^ | 3.30^a^ | 0.459 | <0.0001 | <0.0001 | <0.0001 |
|  | *Collinsella* | -0.02^b^ | -1.13^c^ | 0.03^b^ | -0.06^b^ | -0.14^b^ | 1.31^a^ | 0.148 | 0.4335 | <0.0001 | <0.0001 |
|  | *Coriobacteriaceae_UCG-002* | 0.05 | 0.00 | -0.10 | 0.00 | -0.05 | -0.01 | 0.079 | 0.6283 | 0.5930 | 0.6013 |
|  | *Slackia* | 0.01 | 0.01 | -0.01 | 0.01 | 0.05 | 0.01 | 0.015 | 0.4527 | 0.2005 | 0.1742 |
| Bacteroidota | | 1.36 | 0.02 | 1.27 | 0.84 | -0.66 | 1.05 | 0.663 | 0.9790 | 0.4568 | 0.1076 |
|  | *Alloprevotella* | 0.00^b^ | 0.01^b^ | -0.09^b^ | 0.40^a^ | 0.14^b^ | -0.37^c^ | 0.036 | 0.9861 | <0.0001 | <0.0001 |
|  | *Bacteroides* | 0.94^b^ | 2.60^a^ | 0.81^b^ | 2.82^a^ | -0.77^c^ | -3.58^d^ | 0.325 | 0.2819 | <0.0001 | <0.0001 |
|  | *Muribaculaceae* | 0.95 | 0.07 | 0.07 | 0.41 | -0.23 | -0.46 | 0.444 | 0.4403 | 0.1318 | 0.3442 |
|  | *Parabacteroides* | 0.07^ab^ | 0.00^abc^ | 0.07^ab^ | 0.10^a^ | -0.11^c^ | -0.08^bc^ | 0.033 | 0.8751 | 0.0005 | 0.2062 |
|  | *Prevotella* | 0.04 | 0.08 | 0.05 | 0.02 | -0.03 | -0.09 | 0.019 | 0.2554 | 0.0001 | 0.0697 |
|  | *Prevotellaceae_Ga6A1_group* | 0.02 | 0.00 | -0.05 | 0.00 | 0.12 | 0.00 | 0.010 | 0.2026 | 0.0226 | NA^3^ |
|  | *Rikenellaceae_RC9_gut_group* | -0.02^ab^ | 0.00^ab^ | -0.03^ab^ | 0.05^a^ | -0.01^ab^ | -0.08^b^ | 0.020 | 0.5169 | 0.0505 | 0.0106 |
| Firmicutes | | -1.05 | 6.83 | -0.93 | 3.48 | 0.70 | 6.33 | 1.118 | <0.0001 | 0.1601 | 0.3249 |
|  | *[Eubacterium]_brachy_group* | -0.12^b^ | 0.08^b^ | -0.09^b^ | 2.08^a^ | -0.52^b^ | -2.21^c^ | 0.208 | 0.2091 | <0.0001 | <0.0001 |
|  | *[Eubacterium]_nodatum_group* | 0.02^b^ | 0.00^b^ | 0.17^b^ | 0.48^a^ | -0.12^b^ | -0.63^c^ | 0.038 | 0.0339 | <0.0001 | <0.0001 |
|  | *[Ruminococcus]_gauvreauii_group* | 0.04 | 0.00 | 0.04 | 0.04 | 0.04 | 0.00 | 0.016 | 0.0237 | 0.2350 | 0.2332 |
|  | *[Ruminococcus]_gnavus_group* | -0.10^c^ | 0.04^bc^ | 0.02^bc^ | 0.04^bc^ | 0.29^a^ | 0.10^b^ | 0.039 | 0.7885 | 0.0002 | 0.0019 |
|  | *[Ruminococcus]_torques_group* | -0.11^cd^ | 0.15^a^ | -0.06^bc^ | -0.04^bc^ | 0.06^ab^ | -0.19^d^ | 0.028 | 0.6146 | 0.0190 | <0.0001 |
|  | *Allobaculum* | -0.53^c^ | -4.72^d^ | 1.46^b^ | -0.79^c^ | -0.90^c^ | 5.86^a^ | 0.380 | 0.7399 | <0.0001 | <0.0001 |
|  | *Anaeroplasma* | -0.01^b^ | 0.30^a^ | -0.01^b^ | -0.18^b^ | -0.01^b^ | -0.14^b^ | 0.045 | 0.8926 | 0.0002 | 0.0002 |
|  | *Blautia* | 0.03^b^ | 0.06^b^ | 0.24^ab^ | 0.55^a^ | -0.03^b^ | -0.64^c^ | 0.091 | 0.2425 | <0.0001 | 0.0009 |
|  | *Butyricicoccus* | -0.02 | 0.05 | -0.02 | -0.01 | -0.01 | -0.06 | 0.025 | 0.5682 | 0.1446 | 0.0751 |
|  | *Clostridium_sensu_stricto_1* | -0.60^b^ | 1.08^a^ | -0.30^b^ | -0.37^b^ | -0.83^b^ | -1.95^c^ | 0.222 | 0.3536 | <0.0001 | 0.0002 |
|  | *Dubosiella* | -0.31^c^ | -0.40^c^ | 0.21^ab^ | -0.21^bc^ | -0.28^c^ | 0.32^a^ | 0.100 | 0.7225 | 0.0030 | 0.0007 |
|  | *Enterococcus* | 0.02^b^ | -10.96^c^ | 0.08^b^ | 0.86^b^ | -0.01^b^ | 22.36^a^ | 0.621 | <0.0001 | <0.0001 | <0.0001 |
|  | Erysipelotrichaceae uncultured | -0.95^ab^ | -0.17^a^ | -0.07^a^ | 0.08^a^ | -2.24^b^ | 0.43^a^ | 0.416 | 0.0042 | 0.1188 | 0.0250 |
|  | *Faecalibacterium* | -0.01^bcd^ | -0.01^bc^ | -0.02^cd^ | 0.07^a^ | 0.03^ab^ | -0.06^d^ | 0.011 | 0.7175 | 0.0079 | <0.0001 |
|  | *Faecalibaculum* | -0.11^ab^ | 0.00^a^ | 0.00^a^ | 0.00^a^ | -0.33^b^ | 0.00^a^ | 0.048 | 0.0027 | 0.0135 | 0.0135 |
|  | *Lachnoclostridium* | -0.01 | 0.00 | 0.24 | 0.18 | -0.17 | -0.08 | 0.057 | 0.8577 | 0.0002 | 0.4279 |
|  | Lachnospiraceae unclassified | 0.02^b^ | 0.00^bc^ | -0.12^c^ | 0.40^a^ | -0.01^c^ | -0.65^d^ | 0.048 | 0.2456 | <0.0001 | <0.0001 |
|  | Lachnospiraceae uncultured | -0.05^c^ | 0.00^c^ | 0.14^b^ | 0.32^a^ | -0.10^c^ | -0.34^d^ | 0.030 | 0.8658 | <0.0001 | <0.0001 |
|  | *Lactobacillus* | 0.02^b^ | -0.61^c^ | -0.02^bc^ | 0.72^b^ | 0.01^bc^ | 3.42^a^ | 0.155 | <0.0001 | <0.0001 | <0.0001 |
|  | *Megamonas* | 0.00^b^ | 0.00^b^ | 0.18^a^ | 0.00^b^ | -0.24^c^ | 0.00^b^ | 0.017 | 0.2092 | <0.0001 | <0.0001 |
|  | *Negativibacillus* | 0.21^b^ | 0.00^c^ | 0.00^c^ | 0.47^a^ | -0.23^d^ | -0.49^e^ | 0.044 | 0.9773 | <0.0001 | <0.0001 |
|  | *Oribacterium* | 0.04^bc^ | 0.05^bc^ | 0.32^b^ | 0.92^a^ | -0.16^c^ | -1.06^d^ | 0.072 | 0.0737 | <0.0001 | <0.0001 |
|  | *Peptoclostridium* | 0.54^bc^ | 5.81^a^ | 1.52^b^ | -1.16^c^ | 1.00^bc^ | -3.93^d^ | 0.618 | 0.0807 | <0.0001 | <0.0001 |
|  | *Peptococcus* | 0.10^b^ | 0.00^c^ | 0.04^bc^ | 0.40^a^ | 0.08^b^ | -0.39^d^ | 0.017 | <0.0001 | <0.0001 | <0.0001 |
|  | *Peptostreptococcus* | 1.27^ab^ | 3.72^a^ | 0.00^b^ | 1.22^ab^ | -0.03^b^ | -6.11^c^ | 0.687 | 0.1208 | <0.0001 | <0.0001 |
|  | *Phascolarctobacterium* | 0.01^b^ | 1.13^a^ | 0.05^b^ | -0.20^b^ | -0.03^b^ | -0.76^c^ | 0.047 | 0.2500 | <0.0001 | <0.0001 |
|  | *Romboutsia* | -0.16^bc^ | 0.00^a^ | -0.05^ab^ | 0.00^a^ | -0.22^c^ | 0.00^a^ | 0.029 | <0.0001 | 0.0144 | 0.0144 |
|  | *Sellimonas* | 0.02 | 0.00 | 0.04 | 0.36 | -0.05 | -0.37 | 0.028 | 0.8942 | 0.0011 | NA |
|  | *Streptococcus* | -0.02^b^ | -0.78^c^ | -0.09^b^ | 0.02^b^ | 0.22^b^ | 1.73^a^ | 0.090 | 0.0013 | <0.0001 | <0.0001 |
|  | *Terrisporobacter* | 0.00 | -0.58 | 0.00 | 0.28 | 0.00 | 1.52 | 0.066 | 0.2028 | 0.0227 | NA |
|  | *Turicibacter* | -0.04 | 0.04 | -0.02 | 0.03 | -0.02 | 0.04 | 0.014 | 0.0001 | 0.6195 | 0.3634 |
|  | UCG-005 | 0.08^ab^ | 0.00^b^ | 0.10^ab^ | 0.12^a^ | -0.01^b^ | -0.14^c^ | 0.025 | 0.0063 | <0.0001 | 0.0132 |
| Fusobacteriota | | 0.11 | 0.01 | 1.12 | -0.15 | 0.68 | 0.57 | 0.890 | 0.4670 | 0.7689 | 0.7093 |
|  | *Cetobacterium* | 0.33 | 0.00 | 0.04 | 0.00 | 0.72 | 0.00 | 0.038 | 0.0030 | 0.2832 | NA |
|  | *Fusobacterium* | -1.81^d^ | 25.15^a^ | -4.91^d^ | 3.12^c^ | 7.54^b^ | -27.84^e^ | 0.869 | 0.8437 | <0.0001 | <0.0001 |
| Proteobacteria | | -0.60 | -2.62 | -0.70 | 1.20 | 0.03 | -2.67 | 1.015 | 0.6911 | 0.2026 | NA |
|  | *Anaerobiospirillum* | -0.01^b^ | 0.19^a^ | -0.02^b^ | -0.12^b^ | 0.03^ab^ | -0.07^b^ | 0.034 | 0.8706 | 0.0019 | 0.0013 |
|  | *Escherichia-Shigella* | 0.05^b^ | -15.88^c^ | 0.06^b^ | -3.45^b^ | -0.01^b^ | 14.79^a^ | 0.834 | 0.0421 | <0.0001 | <0.0001 |
|  | *Morganella* | 0.00^c^ | 1.46^a^ | 0.00^c^ | -1.56^d^ | 0.00^c^ | 0.41^b^ | 0.078 | 0.1298 | <0.0001 | <0.0001 |
|  | *Parasutterella* | -0.22 | -0.12 | -0.33 | -0.02 | 0.03 | 0.35 | 0.112 | 0.0208 | 0.0101 | 0.5849 |
|  | *Proteus* | 0.00^b^ | -1.02^d^ | 0.00^b^ | -0.67^c^ | 0.00^b^ | 0.39^a^ | 0.051 | <0.0001 | <0.0001 | <0.0001 |

^1^ABX- = samples collected from dogs prior to antibiotic administration; ABX+ = samples collected after receiving metronidazole (20 mg/kg body weight twice daily) for two wk.
^2^SEM = pooled standard errors of the means.
^3^NA: data not normal, requiring non-parametric statistical analysis.
^a-e^Mean values within a row with unlike superscript letters differ using parametric analysis (P<0.05).

# **Supplementary Figure 1.** Unweighted [A] and weighted [B] principal coordinate analysis plots of in vitro fermentation tubes containing pectin.


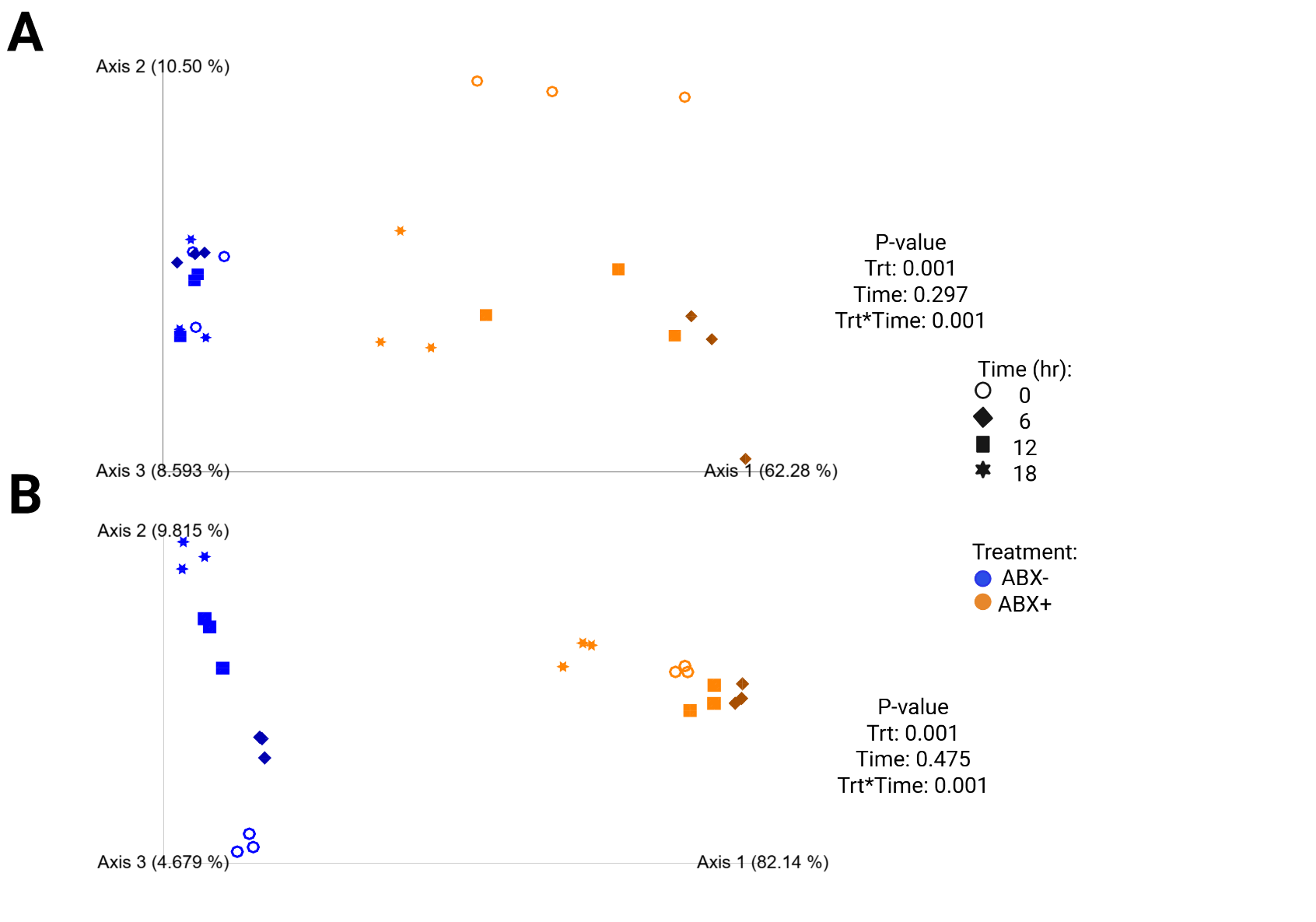


# **Supplementary Figure 2.** Unweighted [A] and weighted [B] principal coordinates analysis plots of in vitro fermentation tubes containing cellulose.

**
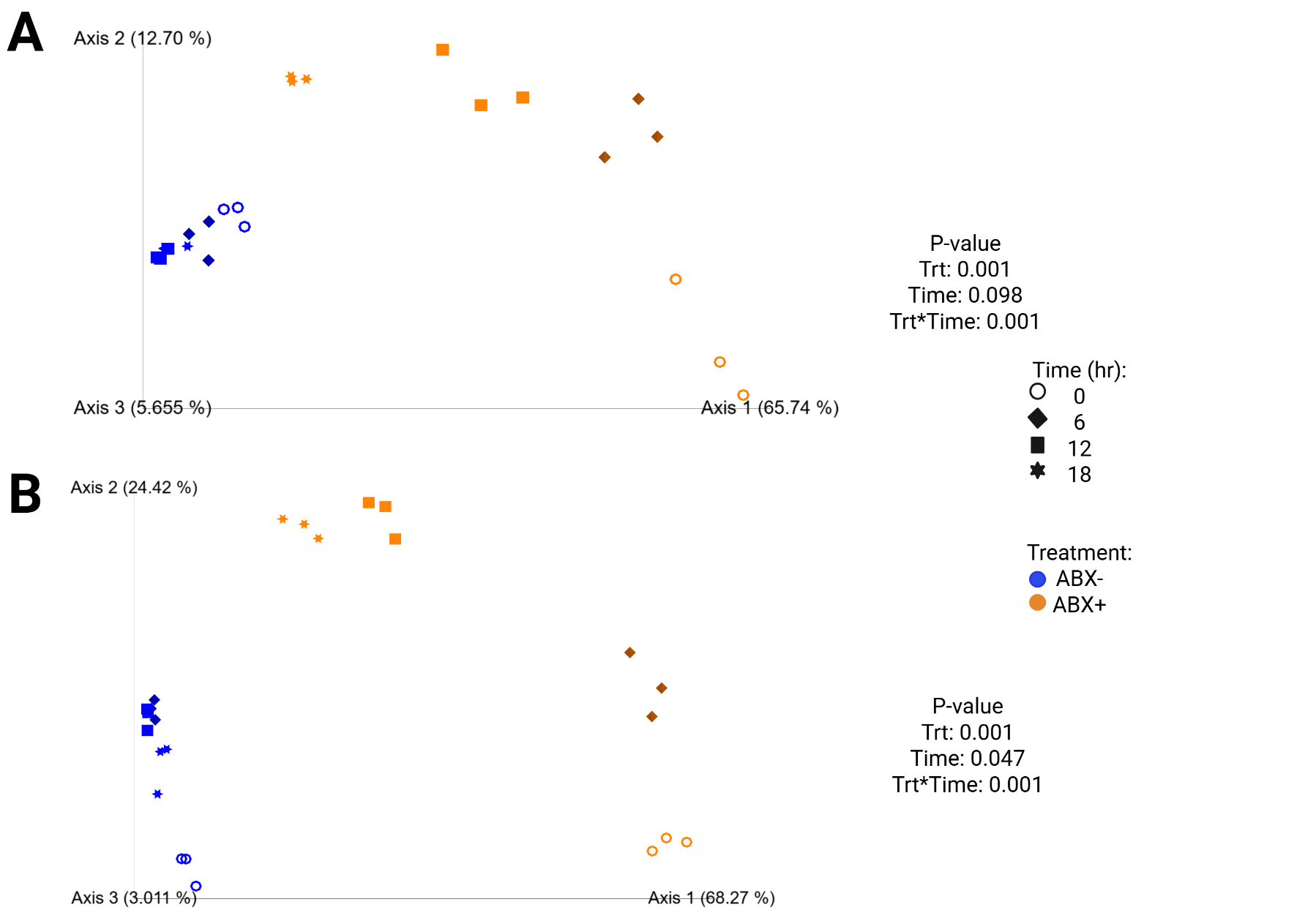
**
